# In silico comparison of SARS-CoV-2 spike protein-ACE2 binding affinities across species and implications for virus origin

**DOI:** 10.1038/s41598-021-92388-5

**Published:** 2021-06-24

**Authors:** Sakshi Piplani, Puneet Kumar Singh, David A. Winkler, Nikolai Petrovsky

**Affiliations:** 1 College of Medicine and Public Health, Flinders University, Bedford Park, 5046 Australia; 2 Vaxine Pty Ltd, 11 Walkley Avenue, Warradale, 5046 Australia; 3 Department of Biochemistry and Genetics, La Trobe Institute for Molecular Science, La Trobe University, Melbourne, VIC 3086 Australia; 4 Monash Institute of Pharmaceutical Sciences, Monash University, Parkville, 3052 Australia; 5 School of Pharmacy, University of Nottingham, Nottingham, NG7 2RD UK

**Keywords:** Computational biology and bioinformatics, Drug discovery, Structural biology, Diseases, Chemistry, Computational chemistry, Small molecules

## Abstract

The devastating impact of the COVID-19 pandemic caused by SARS–coronavirus 2 (SARS-CoV-2) has raised important questions about its origins and the mechanism of its transfer to humans. A further question was whether companion or commercial animals could act as SARS-CoV-2 vectors, with early data suggesting susceptibility is species specific. To better understand SARS-CoV-2 species susceptibility, we undertook an in silico structural homology modelling, protein–protein docking, and molecular dynamics simulation study of SARS-CoV-2 spike protein’s ability to bind angiotensin converting enzyme 2 (ACE2) from relevant species. Spike protein exhibited the highest binding to human (h)ACE2 of all the species tested, forming the highest number of hydrogen bonds with hACE2. Interestingly, pangolin ACE2 showed the next highest binding affinity despite having a relatively low sequence homology, whereas the affinity of monkey ACE2 was much lower despite its high sequence similarity to hACE2. These differences highlight the power of a structural versus a sequence-based approach to cross-species analyses. ACE2 species in the upper half of the predicted affinity range (monkey, hamster, dog, ferret, cat) have been shown to be permissive to SARS-CoV-2 infection, supporting a correlation between binding affinity and infection susceptibility. These findings show that the earliest known SARS-CoV-2 isolates were surprisingly well adapted to bind strongly to human ACE2, helping explain its efficient human to human respiratory transmission. This study highlights how in silico structural modelling methods can be used to rapidly generate information on novel viruses to help predict their behaviour and aid in countermeasure development.

## Introduction

The devastating impact of COVID-19 infections caused by SARS–coronavirus 2 (SARS-CoV-2) has stimulated unprecedented international activity to discover effective coronavirus vaccines and drugs^1–4^. It has also raised important questions, including the mechanisms of zoonotic transfer of these viruses from animals to humans, whether companion animals or those used for commercial purposes can act as infection reservoirs, and why large variations in susceptibility are seen across animal species^5–7^. Understanding how such viruses move between species may help us prevent or minimize similar events in the future. Methods that elucidate the molecular basis for susceptibility differences may also help explain why different human populations exhibit different susceptibilities^8^.

**Figure 1:**
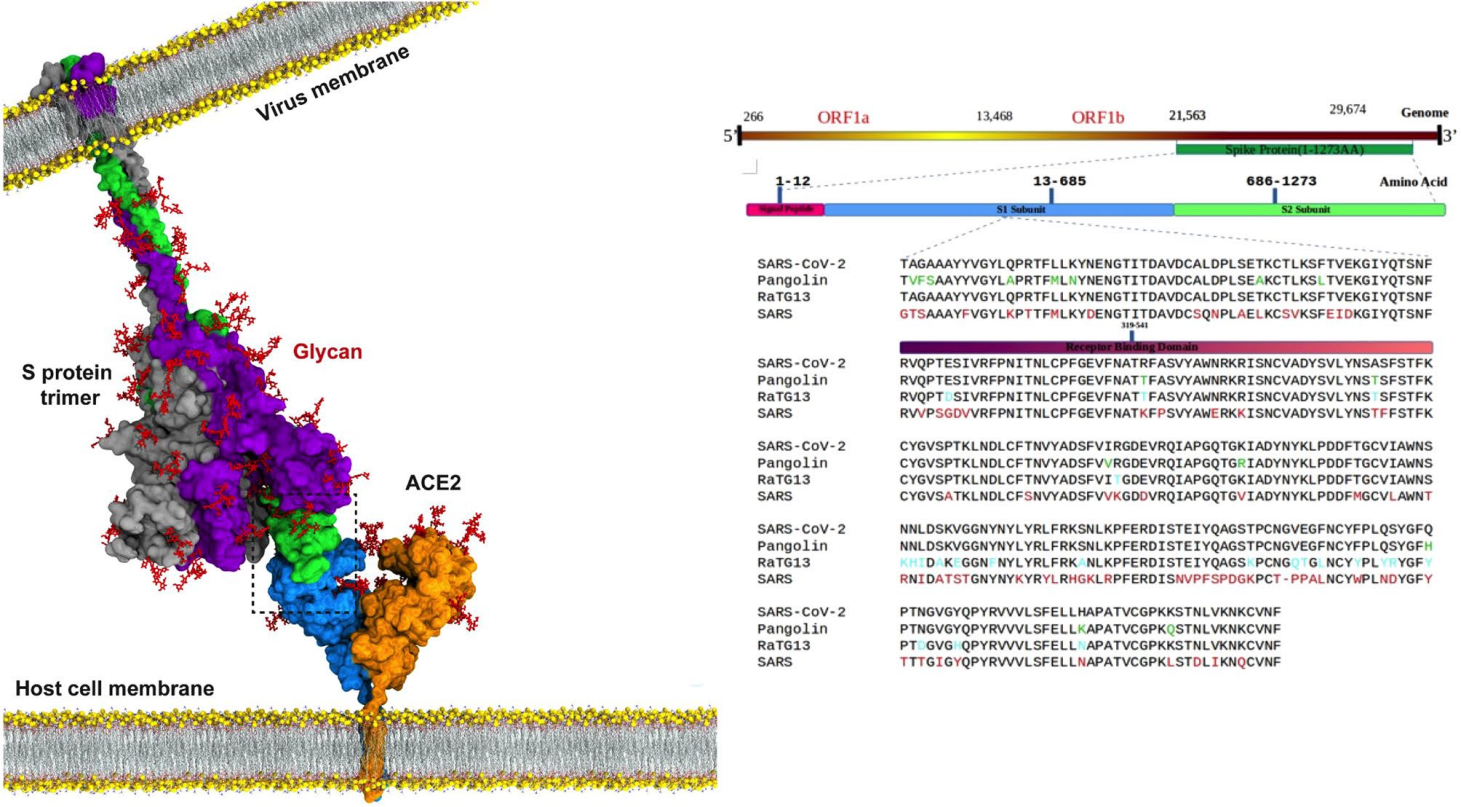
Upper panel: Picture of the complex formed by SARS-CoV-2 S protein and human ACE2 (CC-BY-NC-ND 4.0 International license; adapted from Taka et al.^15^). Lower panel: Sequence alignment of S1 subunit of four closely related spike proteins (sequence commences 60 residues before RBD). Coloured differences in spike protein sequences from pangolin CoV (green), bat RATG13 CoV (cyan) SARS CoV (red) when compared to sequence of SARS-CoV-2. The spike protein receptor binding domain (RBD) is denoted by the red bar in the sequence alignments.

The spike protein (S protein) with its functional polybasic furin cleavage site at the S1–S2 boundary plays a key role in SARS-CoV-2 infectivity^9^. The S protein monomer consists of a fusion peptide, two heptad repeats, an intracellular domain, N-terminal domain, two subdomains and a transmembrane region^10^. Angiotensin converting enzyme 2 (ACE2) was identified as the main receptor for SARS-CoV-2 S protein, mimicking ACE2’s role as the receptor for SARS virus. The binding of S protein to ACE2 is a critical initiating event for infection and human to human transmission (Fig. 1). ACE2 is relatively ubiquitously expressed in humans, being present in the lungs, arteries, heart, kidney, and intestines. ACE2 consists of an N-terminal peptidase M2 domain and a C-terminal collectrin renal amino acid transporter domain.

Non-human species vary markedly in their susceptibility to SARS-CoV-2^7,11,12^. As ACE2 sequences differ between species, this raises the possibility that differences in S protein’s ability to bind ACE2 from different species might underlie species susceptibility to infection. A phylogenetic tree showing ACE2 sequence relatedness across different animal species including bat, pangolin, and snake, all of which had been postulated as a source or intermediate host for SARS-Cov-2^11,13,14^ is shown in Supplementary Fig. 1.

We and others^16–18^ have postulated that structural variation in different species of ACE2 might determine S protein binding and thereby determine which species are permissive to SARS-CoV-2 infection^19^. For example, the low binding affinity of S protein for mouse ACE2 likely explains why mice are not susceptible to SARS-CoV2 infection. Direct measurement of the binding affinity of SARS-CoV-2 S protein to ACE2, e.g., using cell lines transfected with ACE2 proteins from different species, would provide valuable data but is time consuming, and purified or recombinant ACE2 proteins from many relevant animal species were not available at the time our modelling study was performed in early 2020. Hence we show here how an alternative approach using in silico structural modelling and docking algorithms from structure-based drug design was used to determine and compare the binding affinity of SARS-CoV-2 S protein to ACE2 of common and exotic animal species^20–22^. The species studied had either been implicated in transfer of SARS-CoV-2 to humans (e.g., bat, snake pangolin), been reported to be susceptible or resistant to SARS-CoV-2 infection (tiger, mouse, ferret, hamster, civet, monkey) or are important agricultural species or companion animals (cow, horse, cat, dog). The results provided novel insights into the species-specific nature of S protein-ACE2 interaction and predicted which species might be permissive for infection. They may also provide important clues into the origin of the pandemic since the mechanisms for SARS-CoV-2’s appearance in the human population remains unknown despite more than a year passing since the start of the pandemic.

## Results and discussion

### SARS-Cov-2 S protein binds ACE2 of diverse species

The results of docking the receptor binding domains (RBDs) of SARS-CoV-2 S protein and ACE2 of various species using the HDOCK server, refined by MD simulations, are summarized in Tables 1 and 2. The calculated binding energies for the interactions are summarized in Table 2 with the MMPBSA binding energies listed for comparison. These are also presented graphically in Supplementary Fig. 3.

**Table 1:** ACE2 RBD residues interacting with the S protein RBD from MD simulations of complexes.

Species	Accession number	Position																% binding residues shared with hACE2
		19	24	27	28	30	31	34	37	38	41	42	79	83	330	353	393	
*Homo sapiens (human)*	Q9BYF1	S	Q	T	F	D	K	H	E	D	Y	Q	L	Y	N	K	R	100
*Macaca fascicularis (monkey)*	A0A2K5X283	S	Q	T	F	D	K	H	E	D	Y	Q	L	Y	N	K	R	100
*Panthera tigris (tiger)*	XP_007090142.1	S	L	T	F	D	K	H	E	**E**	Y	Q	L	Y	K	K	R	94
*Bos Taurus (cow)*	NP_001019673.2	S	Q	T	F	**E**	K	H	E	D	Y	Q	M	Y	N	K	R	88
*Mesocricetus auratus (hamster)*	A0A1U7QTA1	S	Q	T	F	D	L	Q	E	D	Y	Q	L	Y	N	K	R	88
*Felis catus (cat)*	Q56H28	S	L	T	F	**E**	K	H	E	**E**	Y	Q	L	Y	N	K	R	81
*Rhinolophus sinicus (bat)*	U5WHY8	S	*E*	**M**	F	D	K	T	E	D	H	Q	L	Y	N	K	R	75
*Paguma larvata (civet)*	Q56NL1	S	L	T	F	**E**	K	Y	E	**Q**	Y	Q	L	Y	N	K	R	75
*Equus ferus caballus (horse)*	F6V9L3	S	L	T	F	D	K	S	E	**E**	H	Q	L	Y	N	K	R	75
*Mustela putorius furo (ferret)*	Q2WG88	D	L	T	F	**E**	K	T	E	**E**	Y	Q	-	Y	N	K	R	69
*Canis luparis (dog)*	J9P7Y2	–	L	T	F	**E**	K	Y	E	**E**	Y	Q	L	Y	N	K	R	69
*Mus musculus (mouse)*	Q8R0I0	S	**N**	T	F	**N**	N	Q	E	D	Y	Q	Y	F	N	K	R	63
*Manis javanica (pangolin)*	XP_017505752.1	–	*E*	T	F	**E**	K	S	E	**E**	Y	Q	**I**	Y	N	K	R	63
*Ophiophagus Hannah (snake)*	ETE61880.1	Q	V	K	F	**E**	Q	A	–	D	Y	**N**	N	**F**	N	L	R	38

Residues interacting with the same S residue in different species ACE2, that differ from those in human ACE2, are in bold (conservative replacements), in italics (partially conservative replacements) or in underline (non-conservative replacements).

**Table 2:** Binding free energies of SARS-Cov-2 spike to ACE2 for different species and infection susceptibility reported by other studies.

Species	ΔG_eqn1_ (kcal/mol)	ΔG_MMPBSA_ (kcal/mol)	SARS-Cov-2 infectivity
*Homo sapiens* (human)	− 52.8	− 57.6 ± 0.25	Permissive, high infectivity, severe disease in 5–10%,
*Manis javanica* (pangolin)	− 52.0	− 56.3 ± 0.4	Permissive^23,24^
*Canis luparis* (dog)	− 50.8	− 49.5	Permissive, low/mod infectivity, no overt disease^25,26^
*Macaca fascicularis* (monkey)	− 50.4	− 50.8	Permissive, high infectivity, lung disease^11^
*Mesocricetus auratus* (hamster)	− 49.7	− 50.0	Permissive, high infectivity, lung disease^27,28^
*Mustela putorius furo* (ferret)	− 48.6	− 49.2	Permissive, moderate infectivity, no overt disease^28–30^
*Felis catus* (cat)	− 47.6	− 48.9	Permissive, high infectivity, lung disease^26,29,31^
*Panthera tigris* (tiger)	− 47.3	− 42.5	Permissive, overt disease, RNA positive^26^
*Rhinolophus sinicus* (bat)	− 46.9	− 50.1 ± 1.0	Not permissive^11^
*Paguma larvata* (civet)	− 45.1	− 46.1	No reported infection
*Equus ferus caballus* (horse)	− 44.1	− 49.2	No naturally occurring infections^26^
*Bos taurus* (cow)	− 43.6	− 42.5	No naturally occurring infections^26^
*Ophiophagus hannah* (king cobra)	− 39.5	− 40.7 ± 1.2	No reported infection
*Mus musculus* (mouse)	− 38.8	− 39.4	Resistant to infection^28^

The energies calculated by Eq. 1 and those from the MMPBSA algorithm (see Materials and Methods) were strongly correlated, with r^2^ = 0.76 (Supplementary Fig. 3). The Kendall tau rank correlation for the two methods of calculating binding energies was 0.94. Interestingly, the calculated energies correlated poorly with the degree of ACE2 sequence similarity (r^2^ = 0.27), suggesting ACE2 structural, rather than sequence-based features have a dominant effect on S protein binding. Notably, docking and MD simulation allows binding affinities to be inferred. The 3D arrangement of amino acids determines the binding of two proteins with contribution from a broad range of surface features, including spatial and physicochemical (e.g., electrostatic and lipophilic) features. The present study integrates analysis of these features to determine the predicted binding affinity. Given the large numbers of models involved, with the exception of S protein complexes with human, monkey and pangolin ACE2, we did not analyse the individual molecular interactions contributing to the overall binding energy in each complex. The key interacting residues of ACE2 and spike proteins identified in our study, by inspecting the converged 3D ACE2-S protein complex structure from MD simulations (Table 1), were broadly consistent with other studies^32–34^. Interacting residues were identified using UCSF Chimera ← Find Contacts. Key S protein-interacting residues that were conserved across all species of ACE2 included PHE28, ASN330, LYS353 and ARG357. Other S protein-interacting residues in ACE2, namely TYR41, LYS353, ALA386 and ARG393, were conserved across all ACE2 species except bat, mouse, ferret and pangolin. S protein-interacting residues with King cobra ACE2 were the least conserved with human ACE2, consistent with the low sequence similarity of snake and human ACE2.

### SARS-Cov-2 S protein optimally binds human ACE2

The ability to ascertain which species are permissive to infection could help identify potential intermediate hosts through which the SARS-CoV-2 virus crossed from a speculated bat source to humans. Although the SARS-CoV-2 S protein has only 72% sequence identity to the SARS receptor binding domain (RBD) region, like SARS-CoV-2, human, civet and bat SARS virus all use ACE2 for cellular entry^35–37^. The closest known relative to SARS-CoV-2 identified so far is the bat RaTG13 virus. However, RaTG13 has a different S protein to SARS-CoV-2 as it lacks a polybasic furin cleavage site and has major amino acid differences in its RBD that, at the sequence level, is most similar to the RBD of pangolin CoV (sequence data in Fig. 1). This has led to suggestions that pangolins might have served as the original host of SARS-CoV-2. Notably, our structure-based analysis of the species specificity of SARS-CoV-2 revealed some surprisingly results that differed from those from purely sequence based analyses. Conspicuously, we found that the binding of the SARS-CoV-2 S protein was higher for human ACE2 than any other species we tested, with the ACE2 binding energy order, from highest to lowest, being human > pangolin > dog > monkey > hamster > ferret > cat > tiger > bat > civet > horse > cow > snake > mouse. At its extremes, this ranking accords with experimental observations that humans are highly permissive to SARS-CoV-2 infection whereas mice on the other hand are not susceptible.

### Potential significance of high S protein binding to human ACE2

During the early pandemic, S protein mutations were rare, especially in the RBD region interacting with ACE2. Notably, three mutation sites, V367F, G476S, and V483A were identified within the RBD of some SARS-CoV-2 isolates but only G476S was in the binding interface and its incidence and geographic spread was very small^38^. This minimal S protein RBD mutation during the early pandemic supports the view that the SARS-CoV-2 S protein was already optimally adapted for human ACE2 binding. This finding was surprising as a zoonotic virus typically exhibits the highest affinity initially for its original host species, with lower initial affinity to receptors of new host species until it adapts. As the virus adapts to its new host, mutations are acquired that increase the binding affinity for the new host receptor. Since our binding calculations were based on SARS-CoV-2 samples isolated in China from December 2019, at the very onset of the outbreak, the extremely high affinity of S protein for human ACE2 was unexpected. This high affinity was confirmed in July 2020 by Alexander et al.^39^ who similarly found that the SARS-CoV-2 S protein RBD is optimal for binding to human ACE2 compared to other species. They also commented upon this as a remarkable finding that likely underlies the high transmissibility of SAR-Cov-2 virus among humans. Our results are also consistent with a study that found SARS-CoV-2 RBD bound with much higher affinity to hACE2 through additional hydrogen bonds and hydrophobic interactions when compared to binding of the SARS virus RBD to hACE2^34^.

### Possible role of pangolins as an intermediate host for SARS-CoV-2?

**Figure 2:**
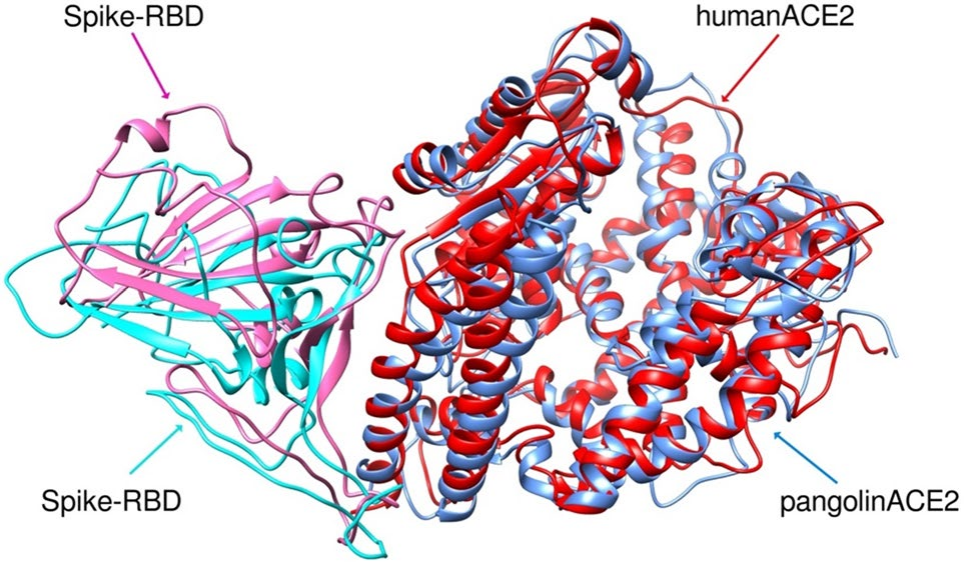
RMSD of overlay of S protein RBD (pink = with pangolin and turquoise = with human) complex with human ACE2 (red) or pangolin ACE2 (blue) after MD simulation showing different geometry of the two complexes.

Interestingly, as shown in Table 1, pangolin and human ACE2 are closest in spike binding energy despite being structurally different and only sharing 10 of 16 interacting residues at the SARS-CoV-2 RBD. The similarity in binding energy is noteworthy as pangolins have previously been imputed as a potential intermediate host to explain the spill-over of a putative bat coronavirus to humans. However, pangolin ACE2 was predicted to have significantly lower binding affinity to S protein than human ACE2 (p = 0.0013). The root-mean-square deviations (RMSD) between the simulated S protein-pangolin ACE2 and S protein-human ACE2 was just 1.211 Å (Fig. 2), indicating the close similarity of these complexes. Most of the pangolin ACE2 differences are conservative replacements of residues in human ACE2 viz., Q24E, D30E, D38E, and L79I, that are likely to make similar contributions to the binding interaction with SARS-CoV-2 spike. Of these, Q24E is only partially conservative because of the change in net charge. Q and E are similar based on the small physicochemical distances, chemical similarity, and experimental exchangeability (Grantham's distance based on composition, polarity and molecular volume; Epstein's coefficient of difference that is based on the differences in polarity and size between replaced pairs of amino acids; Miyata's distance based on volume and polarity; and Experimental Exchangeability devised by Yampolsky and Stoltzfus). E24 also interacts with an Asn residue in the spike RBD suggesting that hydrogen bonding ability rather than salt bridge formation is a more important contribution to the binding energy than electrostatic interactions characterizing salt bridges.

The key differences are the lack of S19 interaction and the replacement of H34 by S34 in pangolin ACE2. However, the MD structures show that the OH moiety in the sidechain of S34 in pangolin ACE2 lies in the same region, and can make similar interactions, as the NH moiety in the imidazole sidechain of H34 in human ACE2. Overall, given the high binding energy of S protein for pangolin ACE2, the possibility of pangolins being an intermediary vector for SARS-CoV-2 cannot be excluded (see also discussion below).

### Exclusion of palm civets as a likely intermediate host for SARS-CoV-2

We were interested to know whether SARS-CoV-2 S protein could bind ACE2 from other species, notably the palm civet, the intermediate host for SARS virus^40^. The predicted binding affinity of SARS-CoV-2 S protein for palm civet ACE2 was low (Δ**G**_**MMPBSA**_** -**46.1, > 10 kcal/mol lower than hACE2), making it extremely unlikely palm civets acted as an intermediate host for SARS-CoV-2.

### Explanation for the large difference in S protein binding to human and monkey ACE2

A surprising observation, not identified by ACE2 sequence analysis, was that the binding affinity of S protein to monkey ACE2 (mACE2) was lower than to human ACE2 by 2.4—6.8 kcal kcal/mol (depending on the binding energy calculation method) despite all 16 S protein binding residues being shared between monkey and human ACE2. This suggested to us that binding energy differences must reside in structural differences rather than these specific residues.

**Figure 3:**
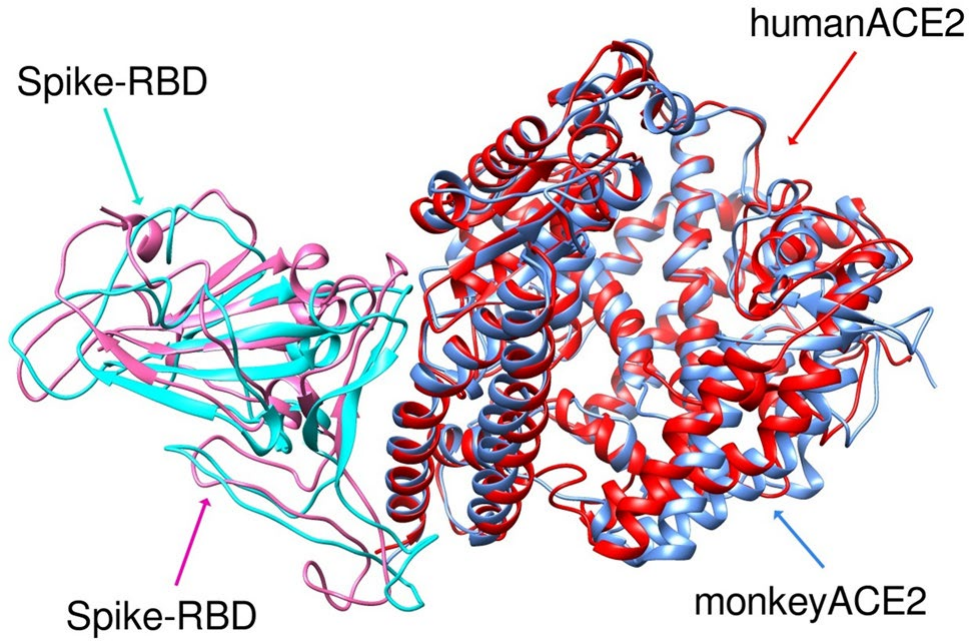
RMSD of overlay of S protein RBD (pink = monkey and turquoise = human) complex with human (red) or monkey (blue) ACE2 after MD simulations showing different geometry of the two complexes.

The structure-based alignment of S protein RBD complexed with hACE2 and mACE2 after MD simulation exhibited an RMSD of 1.359 Å, highlighting differences between the 3-D structures of the two species despite their high sequence similarity (Fig. 3). Although almost all residues in the S protein binding site were common to human and monkey ACE2 sequences (Table 1), structural rearrangements in the S protein and hACE2 binding complex resulted in more H-bonds forming between S protein and hACE2 than the equivalent complex with mACE2. As the binding of S protein to ACE2 is primarily governed by hydrogen bonds and electrostatic interactions, we monitored the total number of intermolecular H-bonds between the S protein and hACE2 and mACE2 proteins throughout the MD simulations. This revealed that the loop comprised of Thr478, Pro479, Cys480, Asn481, Gly482, Val483 and Glu484 plays an important role in the orientation and structural differences of human or monkey ACE2 bound to S protein. Although there is high conservation in the protein sequence of the interacting residues between human and monkey ACE2, MD simulations showed S protein formed more H-bonds with hACE2 (~ 640) than mACE2 (~ 620), thereby explaining the weaker binding affinity of S protein to mACE2, despite the high sequence similarity. Unlike hACE2, mACE2 specifically didn’t form H-bonds with S protein residues Ser19, Phe28 and Lys353. Notably, a stable salt bridge was seen to form between Lys417 in S protein with Asp30 in hACE2 whereas this salt bridge was missing in the complex with mACE2. Lys353 in the S protein also formed an intermolecular salt bridge with Asp38 in hACE2 and was buried in a hydrophobic environment. Hence, although our model supports monkeys being permissive to SARS-CoV-2, it would predict that they should be less susceptible to severe clinical disease than humans. This marries well with experimental data which shows that monkeys are amongst species reported to be susceptible to SARS-CoV-2^5,29,41^, with macaques, hamsters and ferrets being utilised as models of infection^27,30,42^. However, infected young cynomolgus macaques, while they expressed viral RNA in nasal swabs, did not develop overt clinical symptoms with aged animals exhibiting higher viral RNA loads, some weight loss, and moderate interstitial pneumonia and respiratory tract virus replication but ultimately spontaneously clearing the virus without treatment^42,43^.

### SARS-CoV-2 susceptibility for laboratory species

Our model showed hamster ACE2 had high S protein binding, similar to the binding affinity to monkey ACE2. This could be explained by hamster ACE2 sharing 14 out of 16 of the S protein binding residues seen in hACE2. Hence our model predicts hamsters should be permissive to SARS-CoV-2 infection (Table 2). In support of these findings, Syrian hamsters have been shown to exhibit clinical and histopathological responses to SARS-CoV-2 that closely mimic human respiratory tract infections, with high virus shedding and ability to transmit the virus to naïve contact animals^27^. The high susceptibility of hamsters to SARS-CoV-2 infection has made them one of the most utilised small animal models. Our model also predicted that ferret ACE2 has a similar binding strength to the S protein as hamster ACE2 (Table 2). Again, ferrets have been found permissive to SARS-CoV-2 infection, with high virus titre in the upper respiratory tract, virus shedding, acute bronchiolitis and active virus transmission to naïve ferrets through direct contact^29,30^.

At the other extreme, based on the model predicting low S protein binding affinity for murine ACE2, mice should be resistant to infection. Supporting this finding, SARS-CoV-2 has been shown to have inefficient replication in mice^44^. Mice, however, become permissive for SARS or SARS-CoV-2 infection when made transgenic for human ACE2^45^.

### SARS-CoV-2 susceptibility of companion animals

Companion animals are in close contact with their human owners, creating a high risk of cross transmission if these animals were susceptible to SARS-CoV-2 infection. Feline ACE2 (cat and tiger) were shown by our model to have moderate to strong S protein binding affinity. Notably, both these species have now recognised as permissive for SARS-CoV-2 infection. Similarly, our data showed moderate to high affinity of S protein for dog ACE2, predicting dogs should be susceptible to infection. Again, Shen et al. found that that SARS-CoV-2 could be efficiently transmitted to both cats and dogs^46^. Shi et al. reported that ferrets and cats were more permissive to infection than dogs, pigs, chickens, and ducks^29^. Temmam et al. tested 9 cats and 12 dogs living in close contact with their owners with 2 testing positive for SARS-CoV-2 and 11 of 18 others showing clinical signs of COVID-19 but with no serum SARS-CoV-2 antibodies detectable^41^. Interestingly, Goumeniu et al. published an editorial querying the role of dogs in the Lombardy COVID-19 outbreak and recommended use of computational docking experiments, like our own, to provide evidence for or against infection of dogs^47^. Notably, our model predicted that companion animals would be permissive for SARS-CoV-2 infection, with this early prediction being supported by later published data on actual infection of companion animals^48,49^.

### Comparisons of our model predictions to other studies

Supplementary Fig. 5 summarizes the observed and predicted SARS-CoV-2 susceptibilities of species, inferred from analysis of phylogenetic clustering and sequence alignment of ACE2 of species known to be utilized by SARS-CoV-2 virus by Qiu et al.^50^. S protein-ACE2 interaction energies for various species were also predicted by Wu et al. using an automatic docking method, ICM-Pro^51^. However, these energies are inconsistent with the known susceptibilities of the relevant species, likely reflecting the fact that they were calculated based purely on docking calculations with no subsequent MD refinement. Rodrigues et al. published a study that used the HADDOCK docking method to estimate the relative strength of binding affinities of SARS-CoV-2 spike protein for ACE2 proteins of 30 species^52^ with the docking including short restrained MD simulations. As noted by the authors, computational models have limitations, requiring validation against experimental data. For example, their model scored guinea pig and goldfish ACE2 among susceptible species, though it has been shown that guinea pig is non-susceptible^53^, and indirect evidence suggests fish to be naturally resistant. This suggests that longer and potentially more accurate simulation protocols such as the one used in our work, are required to obtain sufficiently precise results to allow accurate comparison of differences in calculated binding energies. Other relevant modelling papers were also published while our paper was under review. Damas et al. published an analysis of ACE2 sequences from 410 vertebrate species, including 252 mammals, generating a probability that they could be used as a receptor by SARS-CoV-2^16^. They classed the species into five risk groups. Man, apes, monkeys, Chinese hamsters, whales and porpoises were included in the high-binding group. Golden hamsters, cattle and cats were members of the medium binding group while dogs, horses and bats were in the low binding group. Notably, pangolins, ferrets, mice, and minks were all assigned to the very low binding group in their analyses, susceptibility predictions that do not correlate well with either our data predictions or with actual experimental observations. Recently, another paper was published by Lam et al.^54^ on the species specificity of S protein-ACE2 interaction. Like us, they used MODELLER to generate ACE2 structures for difference species and selected the best refined model using DOPE scores. They used free energy perturbation methods to calculate the binding energies of SARS-CoV-2 S protein to human and non-human ACE2 proteins although, unlike us, they did not include pangolin ACE2. Conspicuously, they generated energy differences similar to ours, this providing subsequent validation of our computational approach to calculating the relative binding affinities of non-human species. Two additional recent studies have relevance to our study. Spinello et al. published a detailed, microsecond MD simulation of the key molecular interactions driving the higher affinity of SARS-CoV-2 as compared to SARS to hACE2^33^. However, this study did not compare the affinities of S protein for non-human ACE2. Subsequently, Wang and co-workers published a MD study comparing the interactions of SARS-CoV-2 and SARS-CoV spike proteins with the human ACE2 protein with 200 ns simulations^34^ but, again, no other species were considered.

### Implications for the original SARS-CoV-2 animal source

Bats have been suggested as the original host species of SARS-CoV-2 infections in humans. Bat RaTG13 has the highest sequence similarity to SARS-CoV-2 with 96% whole-genome identity, but RaTG13 possesses neither the furin cleavage site nor the pangolin-CoV-like RBD seen in SARS-CoV-2 (Fig. 1, lower panel). Although bats carry many coronaviruses, no evidence of an immediate relative of SARS-CoV-2 in bat populations has been found so far. As highlighted by our data, the binding affinity of SARS-CoV-2 S protein for bat ACE2 is considerably lower than for human ACE2 and other species. Notably, RaTG13 S protein was shown not to bind to human ACE2^55^. Hence even if SARS-CoV-2 did originally arise from a bat precursor virus, which remains unproven, it must have spent considerable time in an intermediate animal host to allow it to adapt its S protein sufficiently to then be able to bind human ACE2. There are currently no explanations for how or where such a transition could have occurred to generate a SARS-CoV-2 spike protein optimised for human ACE2. Evidence of direct human infection by bat coronaviruses is rare, with transmission typically involving an intermediate host. For example, SARS CoV was found to be transmitted from bats to civet cats in which it first adapted before becoming able to infect humans. Hence SARS S protein had to acquire specific mutations to enable each species transition to occur, first to increase its affinity for civet ACE2 and then to increase its affinity for human ACE2. To date, a virus directly related to SARS-CoV-2 has not been identified in bats or any other non-human species, leaving its origins unclear. Wrobel et al. reported a structural biology study of bat RaTG13 and SARS-Cov-2 and calculated the binding affinities of human ACE2 for these viruses^55^. They concluded that, although the structures of SARS-CoV-2 and RaTG13 spike proteins are similar, SARS-CoV-2 spike has a more stable pre-cleavage form, with a k_D_ of 68 ± 9 nM for human ACE2 while RaTG13 bound human ACE2 almost 1000 times more weakly with a k_D_ > 40 µM. They observed that cleavage at the furin site decreased the overall stability of SARS-CoV-2 S protein and fostered the open conformation required for spike to bind to ACE2. They concluded that RaTG13 could not bind effectively to human ACE2 and hence would be unlikely to infect humans.

Early in the COVID-19 outbreak it was suggested that snakes may also be an intermediate vector for SARS-CoV-2. Turtle or snake ACE2 has very low homology to human ACE2 and our model predicted they would have minimal binding to S protein. Subsequently, SARS-CoV-2 was shown to not bind reptile ACE2, excluding snakes as an intermediate host for SARS-CoV-2^14^. As we discussed above, our model predicted pangolin ACE2 to have the closest binding affinity to hACE2, despite a low sequence similarity (86%). In contrast, monkey ACE2 with high similarity (97%) to hACE2 was predicted to have much lower binding affinity to S protein than human or pangolin ACE2. Binding affinities for all other modelled species were all substantially lower than for human ACE2 at the > 99.99% confidence level.

A coronavirus isolated from Malayan pangolins (pangolin-CoV) shared the same S protein RDB sequence as SARS-CoV-2, raising the suggestion that pangolins may have acted as an intermediate SARS-CoV-2 host between bats and humans. Although it has high spike RBD sequence similarity to SARS-CoV-2, pangolin-CoVs are not closely related to SARS-CoV-2, with ~ 90% sequence similarity across their whole genome^23^. It is noteworthy that the RBD common to both pangolin CoV and SARS-CoV-2 binds strongly to both pangolin and human ACE2, despite significant differences in these ACE2 molecules with only 63% of their binding site residues being common (Table 1), and the sequence similarity of ACE2 is only marginally higher between pangolins and human ACE2 ~ 85%, than between and bat and human ACE2 ~ 82%. Remarkably, Pangolin-CoV S protein has 100% amino acid identity with SARS-CoV-2 S protein but has much lower levels of identity of 98.6, 97.8 and 90.7% in the E, M and N proteins^24^. Pangolin CoVs isolated from Malayan pangolins from two different regions in China showed differences in the residues interacting with human ACE2^23^. One possibility might be that a pangolin was simultaneously co-infected with a bat ancestor to SARS-CoV-2 at the same time as being infected by a pangolin CoV. This could have allowed a recombination event to occur whereby the spike RBD of the pangolin CoV was inserted into the S protein of the bat CoV, thereby conferring the bat CoV with high binding for both pangolin and human ACE2. Such recombination events occur with other RNA viruses and explain creation of some pandemic influenza strains^56^. However, such events are rare as they require coinfection of the one host with two viruses at exactly the same time and the SARS-CoV-2 genome was reported to exhibit no evidence of recent recombination, arguing against this possibility^57^. Most importantly, if such a recombination event had occurred in pangolins it would be expected to have triggered an epidemic spread of the new highly permissive SARS-CoV-2-like virus among pangolin populations. Currently there is no evidence of a pangolin SARS-CoV-2 outbreak, making this scenario unlikely. Indeed, pangolins might be predicted to be protected from SARS-CoV-2 infection by the existence of cross-protective neutralising antibodies against pangolin coronaviruses given their close RBD similarity, making it even less likely that a SARS-CoV-2 was widely infecting pangolin populations and indeed no evidence of any such infection has been reported. Notably, all pangolin coronaviruses identified to date lack the furin-like cleavage site between S1/S2 in the SARS-CoV-2 S protein that facilitates its rapid spread through human populations. The fact pangolin CoV S protein does not have the furin cleavage site that is a prominent feature of the SARS-CoV-2 S protein^45^, argues against pangolins being the intermediate vector for transmission of SARS-CoV-2 to humans. The major similarity of SARS-CoV-2 to pangolin-CoV lies in the S protein RBD residues that SARS-CoV-2 acquired by some unknown mechanism.

### Does high human ACE2 binding affinity represent a recent gain-of-function mutation?

Gain of function (GoF) mutations in viruses can lead to pandemics. For example, GoF mutations in influenza virus are associated with mammalian transmissibility, increased virulence for humans, and evasion of existing host immunity^56^. The progressive conditioning and adaptation of a new pandemic virus in humans is well recognized. Phylodynamic analyses of the COVID-19 genomes estimated the date for the most recent common ancestor as late 2019, consistent with the earliest reported date in 2019 for the initial cluster of pneumonia cases in Wuhan^58^. Based on available genome sequence data, this study concluded that the current pandemic has been driven entirely by human-to-human transmission since at least December. As the SARS-CoV-2 structure employed in our studies was obtained from viruses collected early in the outbreak, it is not clear how the very first SARS-CoV-2 strains acquired such a high affinity for human ACE2 without prior exposure. These data suggest that SARS-CoV-2 spike RBD evolved by selection on a human-like ACE2. While pangolin ACE2 has major differences in sequence and structure to hACE2, SARS-CoV-2 binding to pangolin ACE2 is second only to hACE2. Nevertheless, no SARS-CoV-2-like virus has been found in pangolins, suggesting they were not the original source. However, the fact pangolin CoVs can potentially use hACE2 for cell entry indicates that pangolin CoVs are a potential source of future human coronavirus pandemics, particularly if pangolin CoVs were to acquire the SARS-CoV-2 furin cleavage site.

Our study has several limitations, including the use homology models because ACE2 crystal structures for most species were not available, the use of the MMPBSA methods which tend to treat ligand entropy in an approximate manner, intrinsic limitations in the MD methods used (although we used an MD code that is widely recognized as robust), and the possibility that the furin cleavage site and different glycosylation patterns may influence the predicted binding affinities. We consider these issues are minimal as the ACE2 structures for most species are very similar and any deficiencies in the calculation of entropic contributions to the Gibbs free energy of binding and deficiencies in the MD force fields should largely cancel out. The propensity of the S protein RBD to adopt an open conformation is strongly influenced by the presence of the furin cleavage site and by glycosylation (e.g. Casalino et al.^59^). However, the S protein structure used for the binding energy comparisons is the same in all complexes with ACE2 proteins from different species, is derived from the experimental S protein-ACE2 complex structure, and thus is likely to result in cancellation of errors generated by these influences.

Given the seriousness of the ongoing SARS-CoV-2 pandemic, it is imperative that all efforts be made to identify the original source of the virus. It remains to be addressed whether SARS-CoV-2 is completely natural and was transmitted to humans by an intermediate animal vector or whether it might have arisen from a recombination event that occurred in a laboratory handling coronaviruses, inadvertently or intentionally, with the new virus being accidentally released into the local human population. Resolving these questions is of key importance so we can use such information to help prevent any similar outbreak in the future. In summary, our study suggests that from the beginning of this pandemic the SARS-CoV-2 S protein already had very high, optimal binding to hACE2. There is minimal early evidence of selection pressure to further optimise binding, in contrast to what has been seen with other zoonotic viruses at the time of their entry into the human population.

## Materials and methods

### Homology modelling of S protein and ACE2 from multiple species

As no three-dimensional structure of the SARS-CoV-2 S protein was available at the commencement of the project, we generated a homology structure the sequence retrieved from NCBI Genbank Database (accession number YP_009724390.1) in January 2020. A PSI-BLAST search against the PDB database for template selection was performed and the x-ray structure of SARS coronavirus S template (PDB ID 5XLR) was selected with 76.4% sequence similarity to SARS-CoV-2 S protein. The sequence alignment and sequences of related bat and pangolin coronaviruses are shown in Fig. 1.

The protein sequences of the ACE2 proteins for different species and full sequence alignment in are shown in Supplementary Fig. 2. The phylogenetic tree for ACE2 proteins from selected animal species is illustrated in Supplementary Fig. 1. Protein preparation and removal of non-essential and non-bridging water molecules for docking studies and analysis of docked proteins was performed using the UCSF Chimera package (https://www.cgl.ucsf.edu/chimera/).^60^

The 3D-structures of the RBD of SARS-Cov-2 S and non-human ACE2 proteins were built using Modeller 9.23 (https://salilab.org/modeller/),^61^ using the SARS S protein template to generate the homology model of SARS-Cov-2 S. The ACE2 receptors of selected species were similarly homology modelled using the following template structures – 1R42 (human ACE2), 3CSI (human glutathione transferase) and 3D0G (ACE2 structure from spike protein receptor-binding domain from the 2002–2003 SARS coronavirus human strain complexed with human-civet chimeric receptor ACE2) (Supplementary Table 1). Template similarity is important for model building the model; the sequence of *Macaca fascicularis* (monkey, accession number A0A2K5X283) was 97% similar to that of human ACE2 while *Ophiophagus hannah* (king cobra) had a much lower similarity of 61% this template. The quality of the generated models was evaluated using the GA341 score^62^ and DOPE ((Discrete Optimized Protein Energy) method scores^63^, and the models assessed using swiss-model structure assessment server (https://swissmodel.expasy.org/assess).^64^ Structures with the lowest DOPE score were refined by MD simulations (vide infra) and used for further analysis. We generated 10 homology models per protein that were refined and optimized in gromacs.

The modelled structures were also assessed for quality control using Ramachandran Plot and molprobity scores in SWISSModel. The Ramachandran plot checks the stereochemical quality of a protein by analysing residue-by-residue geometry and overall structure geometry and visualizing energetically allowed regions for backbone dihedral angles ψ against φ of amino acid residues in protein structure. The Ramachandran score of SARS-CoV-2 spike protein was 90% in the binding region and the molprobity score was 3.17. The Ramachandran score of the percentage of amino acid residues in the various species ACE2 that fall into the energetically favoured region ranged from 96–99% (Supplementary Table 2). Notably, there were no Ramachandran outliers in the RBD. The predicted ACE2 structures and Ramachandran plots for each species are summarized in Supplementary Fig. 4. The molprobity provides evaluation of model quality at both the global and local level, this combines protein quality score that reflects the crystallographic resolution of a model^65^. It is a log-weighted combination of the number of serious atom clashes per 1000 atoms, percentage Ramachandran not favoured, and percentage bad side-chain rotamers. A good molprobity score is one that is equal to or lower than the crystallographic resolution. For reference, the MolProbity score for the x-ray structures of the templates (PDB IDs) were: 1R42 = 3.01; 3SCI = 3.14; 3D0G = 2.74 and 6M17 = 1.99. The Ramachandran and Molprobity scores show that all the built structures were of good quality and suitable for use in further studies.

**Figure 4:**
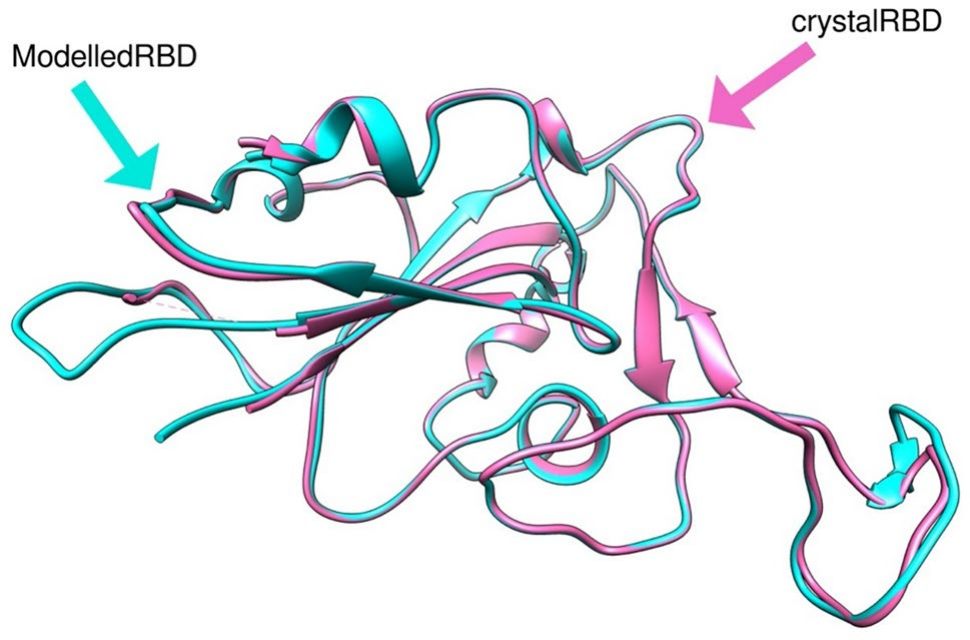
3D structure of SARS-CoV-2 S protein (PDB ID 6M0J (open form) and the homology modelled structure from Modeller. This demonstrates the very high structural similarity of our homology modelled spike protein structure with the EM structures (PDB ID 6M0J (RBD)), with an RMSD of 0.36 Å.

The structure of the open form of the SARS-Cov-2 S protein was published subsequently (e.g., PDB ID 6VYB)^66^. Figure 4 shows the very high structural similarity of our homology modelled spike protein structure with the EM structures (PDB ID 6M0J (RBD) and 6VYB (open state)) with RMSD of 0.36 Å.

### Docking of SARS-Cov-2 S protein with ACE2 proteins

These homology modelled ACE2 structures were docked against SARS-CoV-2 S protein structure using a state-of-the-art package HDOCK (http://hdock.phys.hust.edu.cn/).^67,68^ This performs rigid-body docking by mapping the receptor and ligand molecules onto grids. It docks two molecules using an FFTW-based hierarchical approach. First, possible binding modes are globally sampled through an FFT-based global search strategy with an improved shape complimentary scoring method. Specifically, one molecule is fixed, and second molecule is rotated and translated in space. For each movement of the ligand, both the receptor and ligands molecules are mapped onto grids that extend past the proteins that account for long-range interactions of atoms. Molecular docking was performed on the homology modelled SARS-CoV-2 S protein and human and animal ACE2 proteins using the hybrid docking method because attempts to use the template-free docking method with the structures generated by Modeller were unsatisfactory. The hybrid method used the structural template for the complex (PDB ID 6M17) to generate the results reported here. All docking poses were ranked using an energy-based scoring function.

The hybrid docking procedure may potentially introduce bias into the structures of the S protein bound to ACE2 from non-human species because it uses a human ACE2 complex x-ray structure as a template. To check for possible bias, the ACE2 structures generated by HDOCK were compared with those generated independently by Modeller. The Cα backbones of the ACE2 structures aligned with RMSD values between 0.5 and 0.8 Å (see Supplementary Table 3), exhibiting very strong structural similarities. Additionally, complexes were subjected to molecular dynamics simulation to wash out any template-induced bias.

### Molecular dynamics simulation of docked complexes

The final docked SARS-Cov-2 spike/ACE2 protein complexes were optimized using the AMBER99SB-ILDN force field in gromacs2020 (http://www.gromacs.org/).^69^ Simulations were carried out using the GPU accelerated version of the program and implementing periodic boundary conditions in ORACLE server. The final docked structures were selected by cluster analysis of the docked conformation and based on the RMSD analysis of docked conformation of our structures with 3D0G (SARS-RBD and ACE2). Docked complexes were immersed in a truncated octahedron box of TIP3P water molecules. The solvated box was further neutralized with Na + or Cl − counter ions using the tleap program. Particle Mesh Ewald (PME) was employed to calculate the long-range electrostatic interactions. The cut-off distance for the long-range van der Waals (VDW) energy term was 12.0 Å. The system was minimized without restraints. We applied 2500 cycles of steepest descent minimization followed by 5000 cycles of conjugate gradient minimization. After system optimization, the MD simulations was initiated by gradually heating each system in the NVT ensemble from 0 to 300 K for 50 ps using a Langevin thermostat with a coupling coefficient of 1.0/ps and with a force constant of 2.0 kcal/mol·Å2 on the complex. Finally, a production run of 100 ns of MD simulation was performed under a constant temperature of 300 K in the NPT ensemble with periodic boundary conditions for each system. During the MD procedure, the SHAKE algorithm was applied to all covalent bonds involving hydrogen atoms. The time step was 2 fs. The structural stability of the complex was monitored by the RMSD and RMSF values of the backbone atoms of the entire protein. Finally, the free energies of binding were calculated for all simulated docked structures.

Calculations were also performed for up to 500 ns to ensure that 100 ns is sufficiently long for convergence and that the docked conformation and protein–protein interaction was stable. We ran simulation of our docked spike RBD-human ACE2 for 500 ns and confirmed convergence by RMSD and RMSF. All complexes stabilized during simulations, with RMSD fluctuations converging to a range of 0.5 to 0.8 nm. We found that the complex had stabilised after 50 ns so considered that 100 ns was an adequate simulation time. The RMSD values for superimposition of the Cα backbones of each ACE2 structure before and after 100 ns simulation was 1.2 ± 0.1 Å, showing movement away from the initial HDOCK structures. We analysed the RMSF graph for the protein and did not observe much fluctuation in the amino acids. We used three production runs with different random starting seeds to estimate binding energies and binding energy uncertainties for each of the strongest binding ACE2 structures – human, bat, and pangolin. The binding energies in the Table 2 are based on a 10 ns analysis Sect. (1000 frames).

We also compared 100 ns simulated structures of the ACE2 proteins from all species against those generated by homology modelling (most x-ray structures were not available) and found the RMSD values for Cα alignments between 0.5 and 0.8 Å. This suggests that any memory of the human template has been removed or minimized. We also compared the structures generated independently by homology (Modeller) and HDOCK (Supplementary Table 3) and they agreed very well (RMSD < 1 Å).

### Calculation of binding free energies of complexes

The binding free energies of the protein‐protein complexes were evaluated in two ways. The traditional method is to calculate the energies of solvated SARS-CoV-2 S and ACE2 proteins and that of the bound complex proteins and derive the binding energy by subtraction.

1 $$ \Delta {\text{G }}\left( {{\text{binding}},{\text{ aq}}} \right) = {\text{ G }}\left( {{\text{complex}},{\text{ aq}}} \right){-}{\text{G }}\left( {{\text{spike}},{\text{ aq}}} \right){-}{\text{G }}\left( {{\text{ACE2}},{\text{ aq}}} \right) $$

We also calculated binding energies using the molecular mechanics Poisson Boltzmann surface area (MM-PBSA) tool in Gromacs that is derived from the nonbonded interaction energies of the complex^70,71^. The method is also widely used method for binding free energy calculations. The binding free energies of the protein complexes calculated using the most stable 10 ns section of the equilibrium phase from the output files of the 100 ns MD simulations. The g_mmpbsa tool in Gromacs was used after molecular dynamics simulations. It uses a number of MD snapshots to calculate free binding energy. We analysed the RMSD plot of all the complexes to understand the convergence of the MD trajectory. We identified the region where the complexes were most stable for 10 ns, then frames were extracted from this part of the trajectory to calculate the free binding energy using VdW, electrostatic, polar, and non-polar solvation energy. The output files obtained were used to post-process binding free energies by the single-trajectory MM-PBSA method. Specifically, for a non-covalent binding interaction in the aqueous phase the binding free energy,

2 $$ \Delta {\text{G }}\left( {{\text{bind}},{\text{aqu}}} \right) = \Delta {\text{G }}\left( {{\text{bind}},{\text{vac}}} \right) + \Delta {\text{G }}\left( {{\text{bind}},{\text{solv}}} \right) $$

where ΔG (bind,vac) is the binding free energy in vacuum, and ΔG(bind,solv) is the solvation free energy change upon binding:

3 $$ \Delta {\text{G }}\left( {{\text{bind}},{\text{solv}}} \right) = {\text{ G }}\left( {{\text{R}}{:}{\text{L}},{\text{ solv}}} \right) - {\text{ G }}\left( {{\text{R}},{\text{solv}}} \right) - {\text{ G }}\left( {{\text{L}},{\text{solv}}} \right) $$

where G (R:L,solv), G (R,solv) and G (L,solv) are solvation free energies of complex, receptor and ligand, respectively^72^.

Free energy decomposition analyses were also performed by MM-PBSA decomposition to get a detailed insight into the interactions between the ligand and each residue in the binding site. The binding interaction of each ligand–residue pair includes three terms: the van der Waals contribution, the electrostatic contribution, and the solvation contribution.

As the simulations are very lengthy, we only ran multiple simulations for the species proposed as intermediate hosts and potential sources of the original virus, human, pangolin, bat and snake to estimate the uncertainty in the binding energies. As the ACE2 proteins for all species were extremely similar in sequence, we expected that these simulation error estimates would be of the same order for all other species. The predicted binding energies for these other species were significantly lower than those of human ACE2. We also used a statistical test to calculate the probability of the pangolin and human ACE2 affinities being different.

## Supplementary Information

Supplementary Information.

## Data Availability

The coordinates of the S protein-ACE2 complexes will be deposited in. data repositories at La Trobe University and Flinders University.
