# Supplementary material for: In silico comparison of SARS-CoV-2 spike protein-ACE2 binding affinities across species and implications for virus origin
Source: Sci Rep. 2021 Jun 24;11:13063. doi: 10.1038/s41598-021-92388-5 (PMC8225877; doi:10.1038/s41598-021-92388-5)
Supplement: Supplementary file 1 — Supplementary Information. [file 41598_2021_92388_MOESM1_ESM.docx]

**In silico comparison of SARS-CoV-2 spike protein-ACE2 binding affinities across species and implications for viral origin**

Sakshi Piplani^1,2^, Puneet Kumar Singh^2^, David A. Winkler^3-6^*, Nikolai Petrovsky^1,2^*

^1^ College of Medicine and Public Health, Flinders University, Bedford Park 5046, Australia

^2^ Vaxine Pty Ltd, 11 Walkley Avenue, Warradale 5046, Australia

^3^ La Trobe University, Kingsbury Drive, Bundoora 3086, Australia

^4^ Monash Institute of Pharmaceutical Sciences, Monash University, Parkville 3052, Australia

^5^ School of Pharmacy, University of Nottingham, Nottingham NG7 2RD. UK

^6^ CSIRO Data61, Pullenvale 4069, Australia

**SUPPLEMENTARY INFORMATION**

**Supplementary Table 1**. Human template structures used to model selected ACE2 species and similarity scores of each ACE2 sequence to the selected template used.

| **Species** | **Accession No.** | **Database** | **Template** | **Similarity** |
| --- | --- | --- | --- | --- |
| *Rhinolophus sinicus* (Bat) | AGZ48803.1 | UniProt | 3SCI | 79.73% |
| *Mus musculus* (Mouse) | Q8R0I0 | UniProt | 1R42 | 84.27% |
| *Mustela putorius furo* (Ferret) | Q2WG88 | UniProt | 1R42 | 83.44% |
| *Mesocricetus auratus* (Hamster) | A0A1U7QTA1 | UniProt | 1R42 | 87.58% |
| *Felis catus* (Cat) | Q56H28 | UniProt | 1R42 | 85.93% |
| *Canis luparis* (Dog) | J9P7Y2 | UniProt | 1R42 | 84.93% |
| *Paguma larvata* (Ccivet) | Q56NL1 | UniProt | 3D0G^†^ | 86.77% |
| *Macaca fascicularis* (Monkey) | A0A2K5X283 | UniProt | 1R42 | 96.91% |
| *Manis javanica* (Pangolin) | XP_017505752.1 | NCBI | 1R42 | 85.57% |
| *Ophiophagus hannah (King cobra)* | V8NIH2 | UniProt | 1R42 | 61.42% |
| *Equus caballus (Horse)* | F6V9L3 | UniProt | 6MI7 | 85.91% |
| *Panthera tigris altaica (Tiger)* | XP_007090142.1 | NCBI | 6MI7 | 85.91% |
| *Bos taurus (Cow)* | NP_001019673.2 | NCBI | 6MI7 | 80.30% |

**†** ACE2 structure from spike protein receptor-binding domain from the 2002-2003 SARS coronavirus human strain complexed with human-civet chimeric receptor ACE2

**Supplementary Table 2.** MolProbity and Ramachandran scores for ACE2 modelled structures for selected species

| **Species** | **MolProbity Score** | **Ramachandran Score**  **(favoured region)** | **Ramachandran Outliers** |
| --- | --- | --- | --- |
| *Rhinolophus sinicus (bat)* | 2.45 | 97.8% | 0.18% |
| *Mus musculus (mouse)* | 2.49 | 98.2% | 0.16% |
| *Mustela putorius furo (ferret)* | 2.5 | 98.3% | 0.17% |
| *Mesocricetus auratus (hamster)* | 2.59 | 98.7% | 0.00% |
| *Felis catus (cat)* | 2.42 | 98.5% | 0.33% |
| *Paguma larvata (civet)* | 2.52 | 97.3% | 0.34% |
| *Macaca fascicularis (monkey)* | 2.59 | 98.2% | 0.34% |
| *Manis javanica (pangolin)* | 2.42 | 98.3% | 0.17% |
| *Ophiophagus hannah (king cobra)* | 2.93 | 96.0% | 0.58% |
| *Canis lupus familiaris (dog)* | 2.9 | 97.1% | 0.98% |
| *Equus caballus (horse)* | 2.29 | 96.3% | 0.55% |
| *Panthera tigris altaica (tiger)* | 2.25 | 96.6% | 0.14% |
| *Bos Taurus (cattle)* | 2.70 | 96.2% | 0.56% |

**Supplementary Table 3**. Column 2 shows RMSD for alignments of Cα backbones RMSD for modelled ACE2 with its corresponding ACE2 in the HDOCK generated ACE2-S protein complexes, Column 3 shows values of the all-atom RMSD for modelled ACE2 with its corresponding ACE2 in the HDOCK generated ACE2-S protein complexes

| **Species** | **RMSD Å Cα ACE2** | **RMSD Å complex** |
| --- | --- | --- |
| Bat | 0.82 | 0.88 |
| Cat | 0.54 | 0.85 |
| Cattle | 0.77 | 0.79 |
| Civet | 0.74 | 0.85 |
| Dog | 0.69 | 0.82 |
| Ferret | 0.59 | 0.84 |
| Hamster | 0.60 | 0.85 |
| Horse | 0.82 | 0.84 |
| Monkey | 0.56 | 0.85 |
| Pangolin | 0.54 | 0.86 |
| Snake | 0.84 | 0.89 |
| Tiger | 0.87 | 0.85 |
| Mouse | 0.54 | 0.86 |


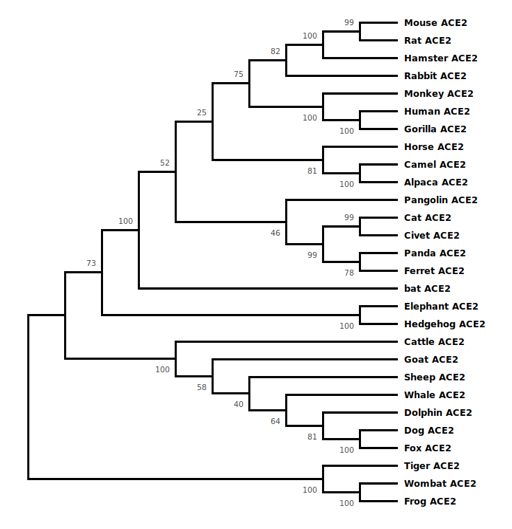


**Supplementary Figure 1**. Phylogenetic tree showing relatedness of sequences of ACE2 proteins from selected species.


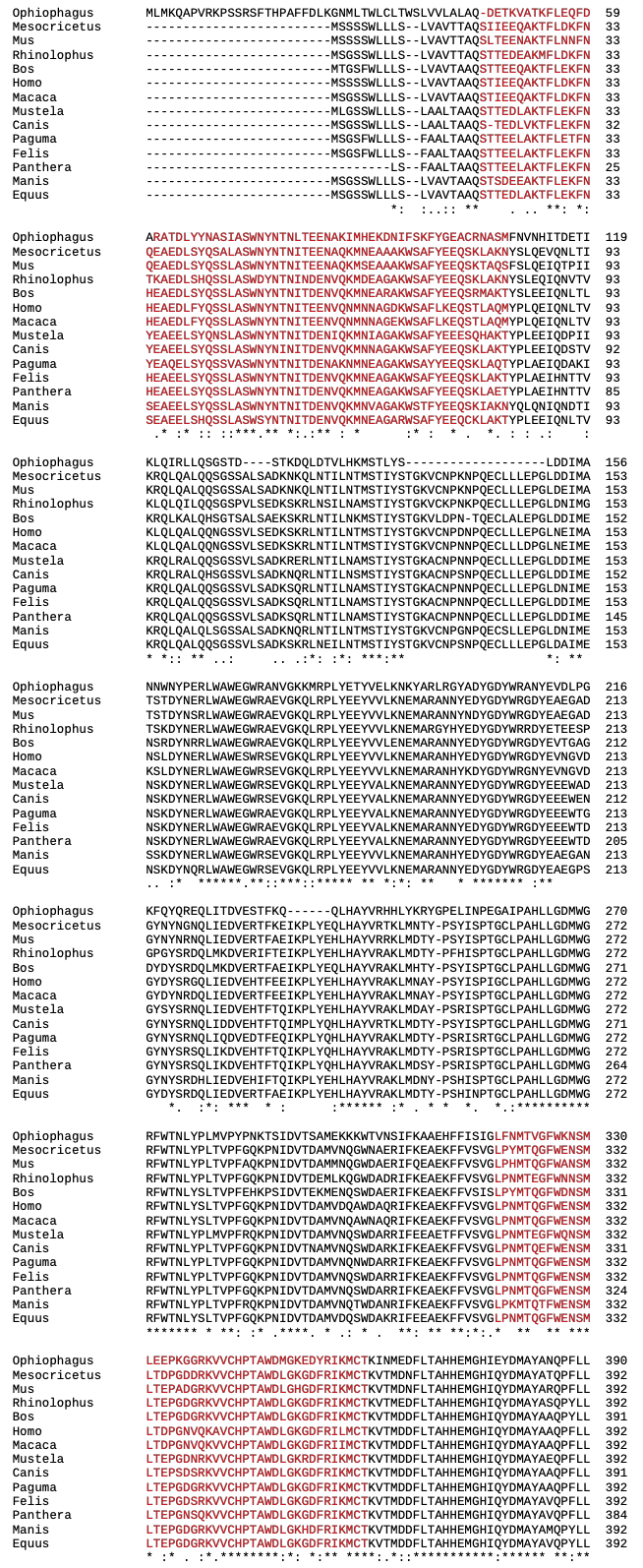


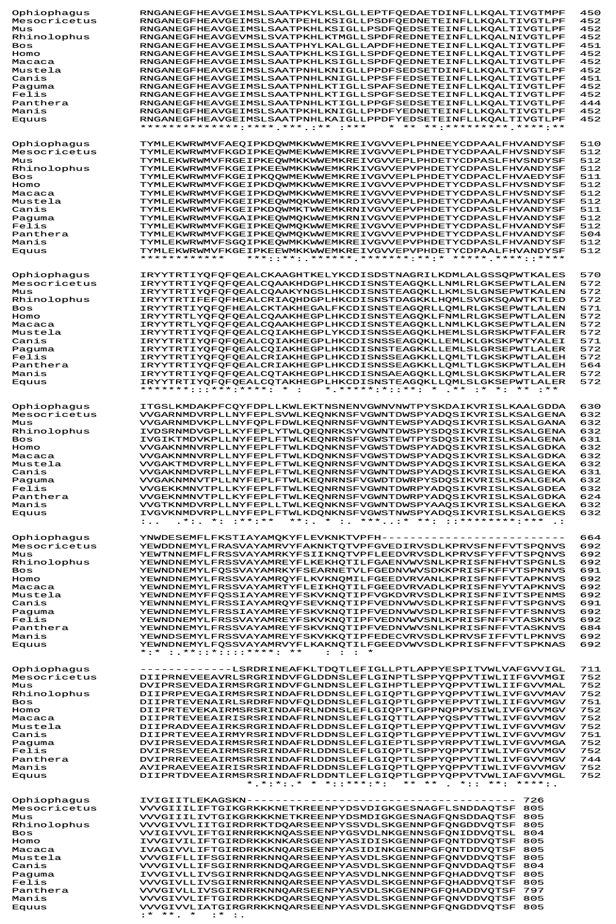


**Supplementary Figure 2**. Sequence alignment of ACE2 amino acid sequence from selected species. The SAR-Cov-2 spike protein binding region is highlighted in red.


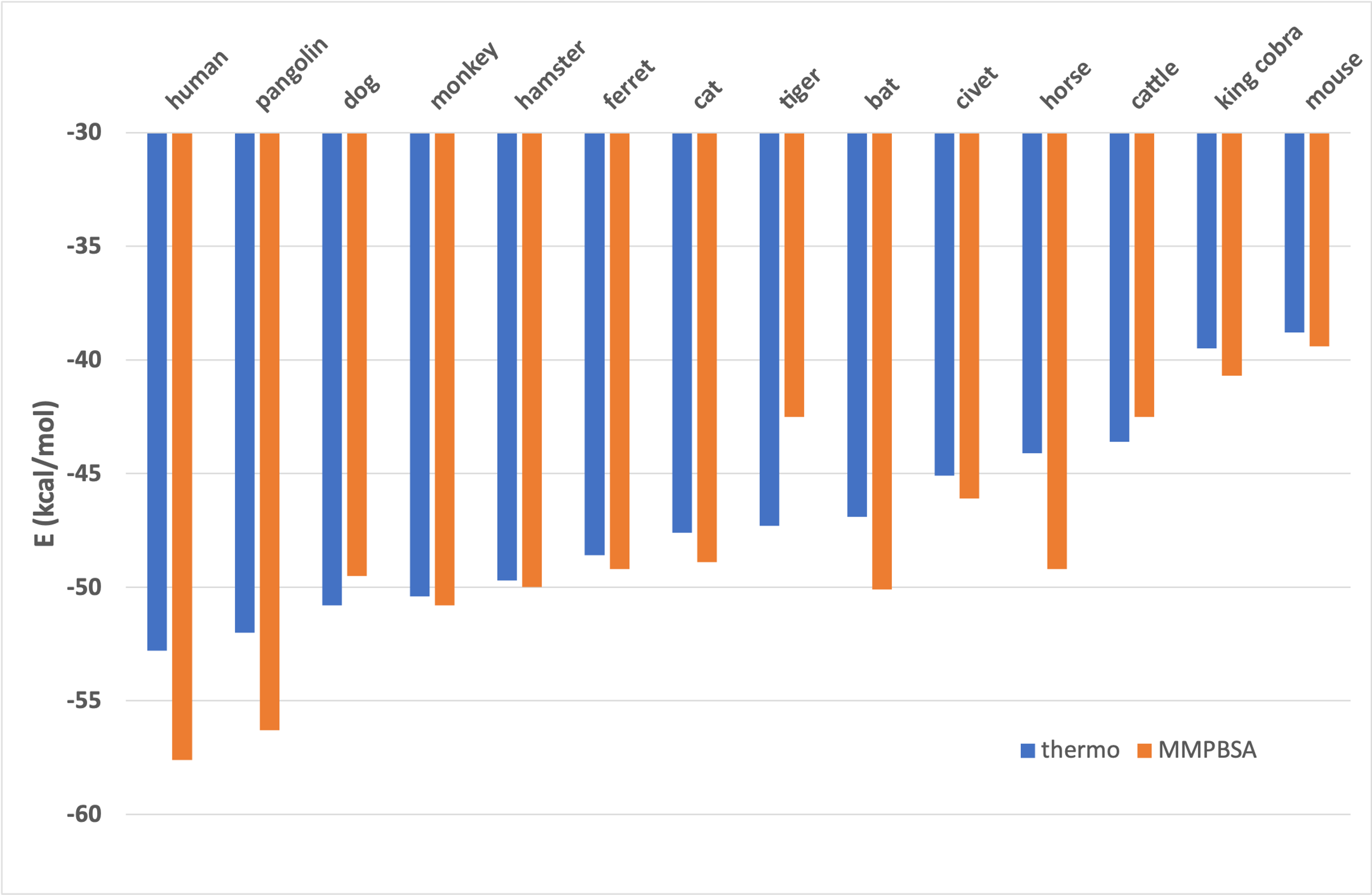


**Supplementary Figure 3**. Bar chart showing the relative binding affinities of ACE2 from various species to the SARS-CoV-2 spike protein RBD.

**BAT ACE2**

**
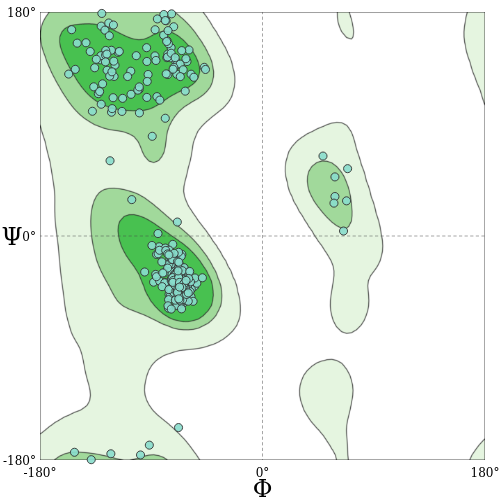
**


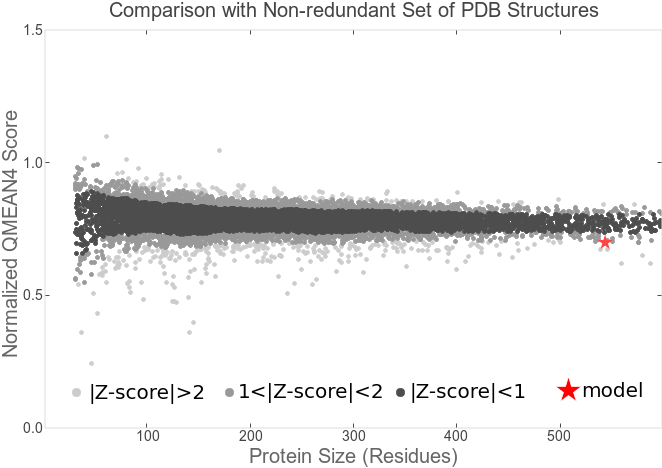

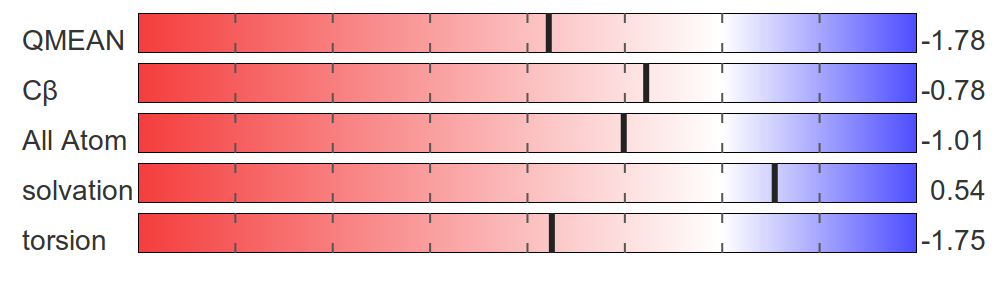

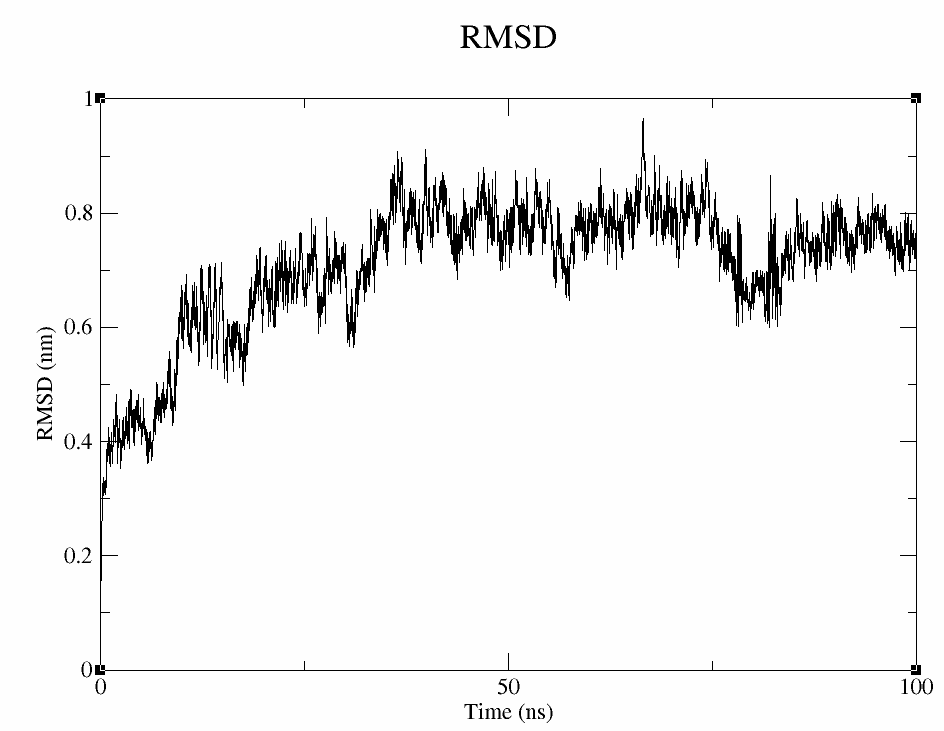

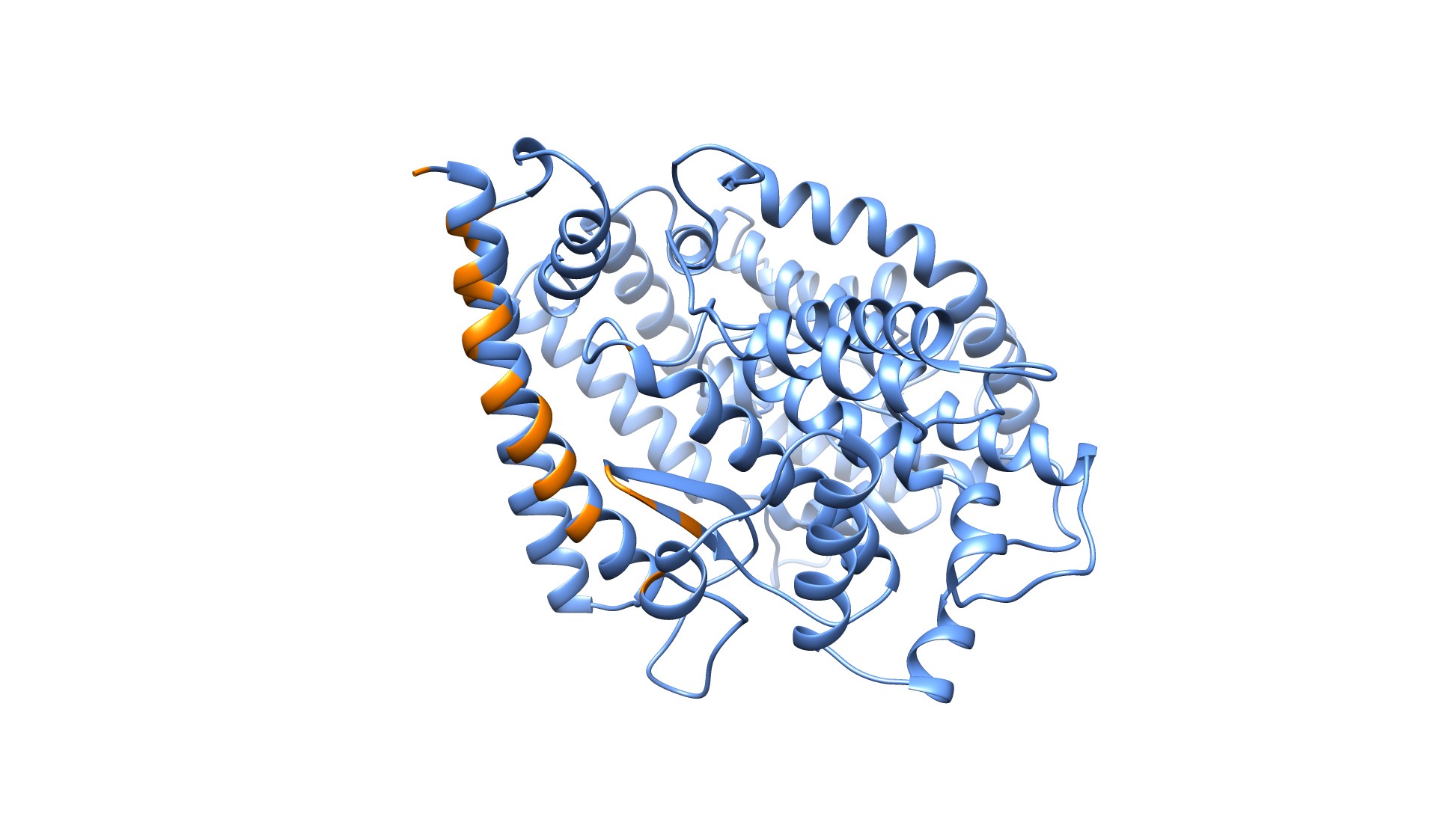


**CAT ACE2**


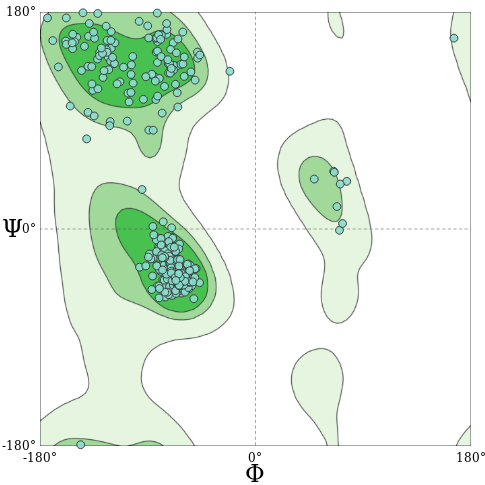

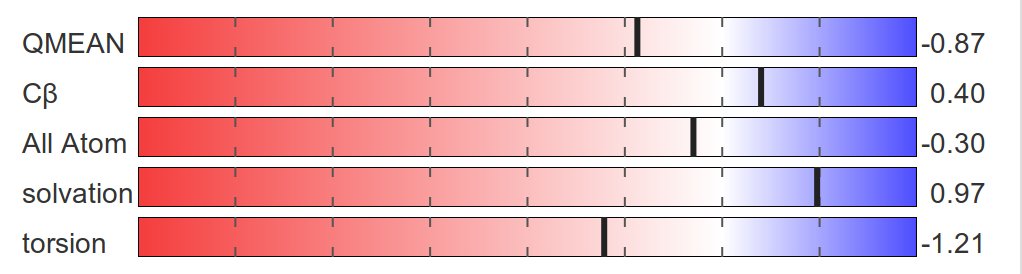

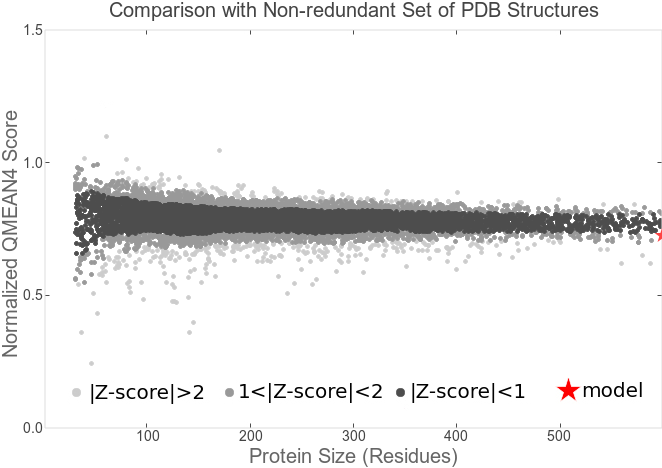

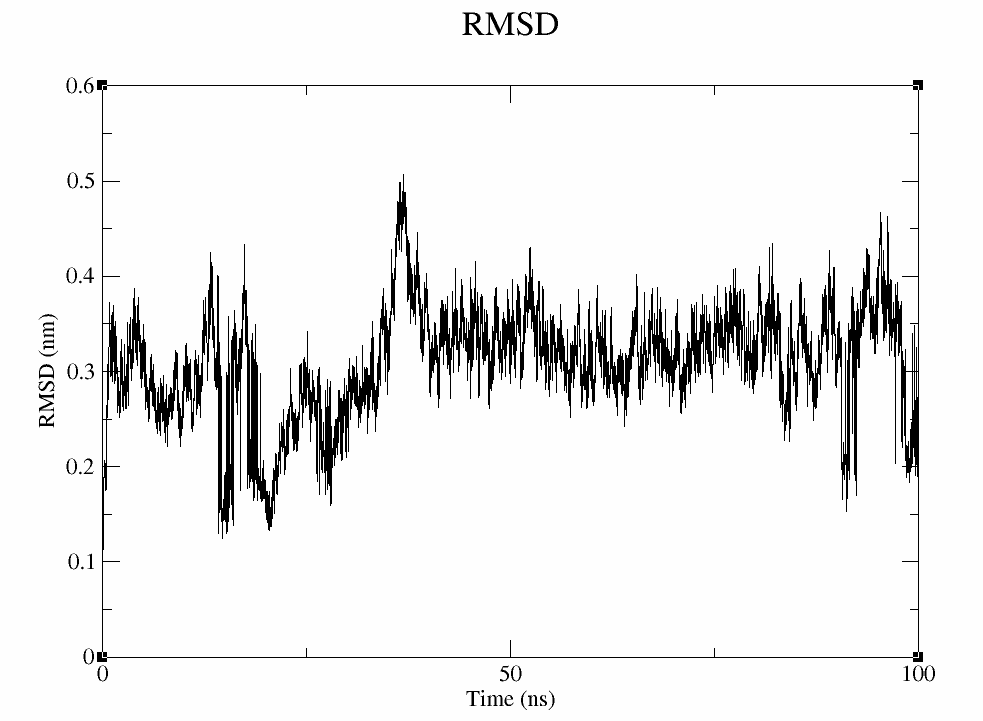

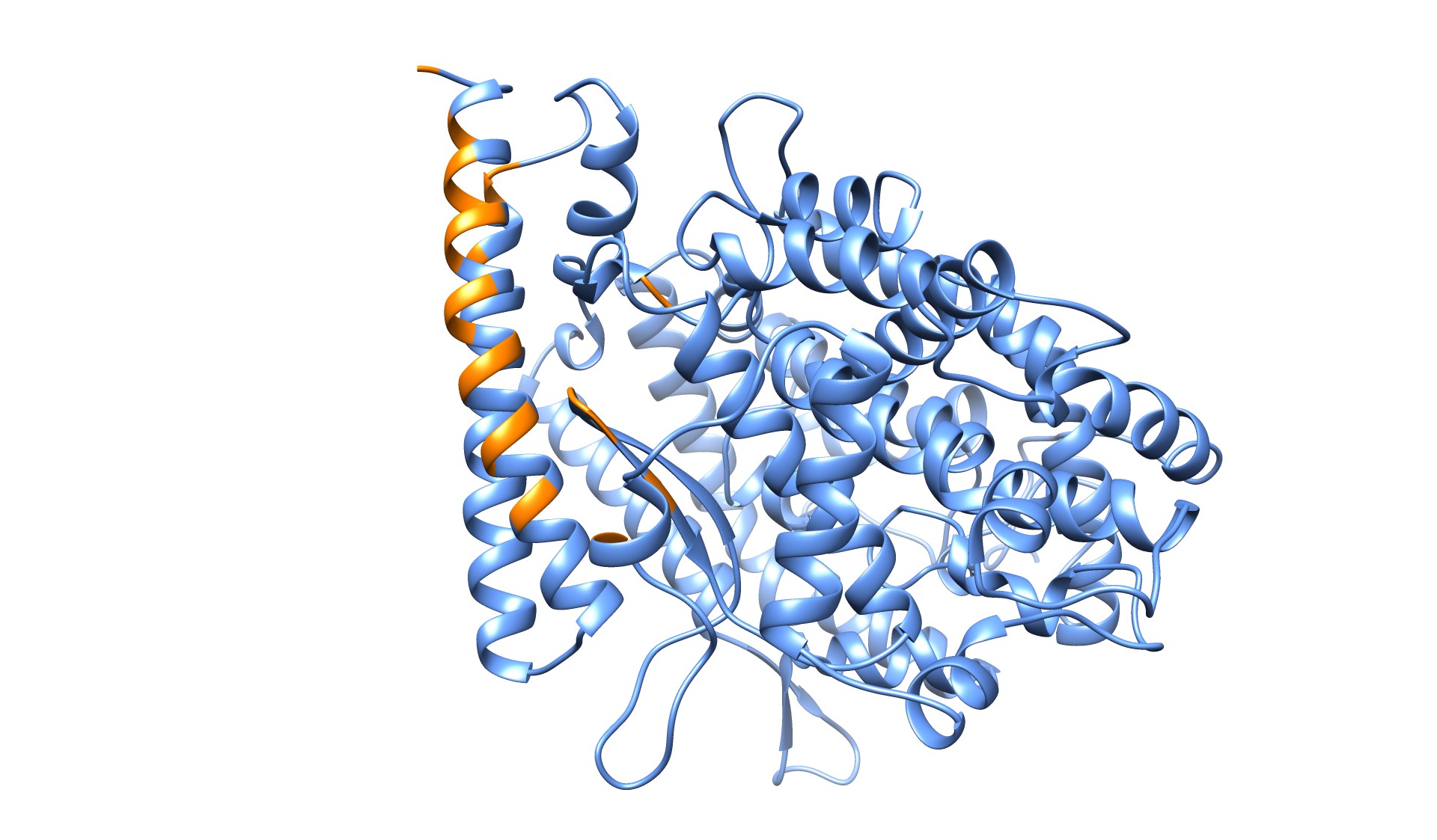


**CIVET ACE2**


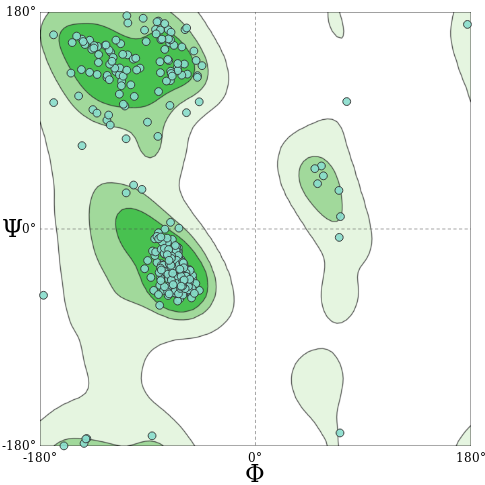

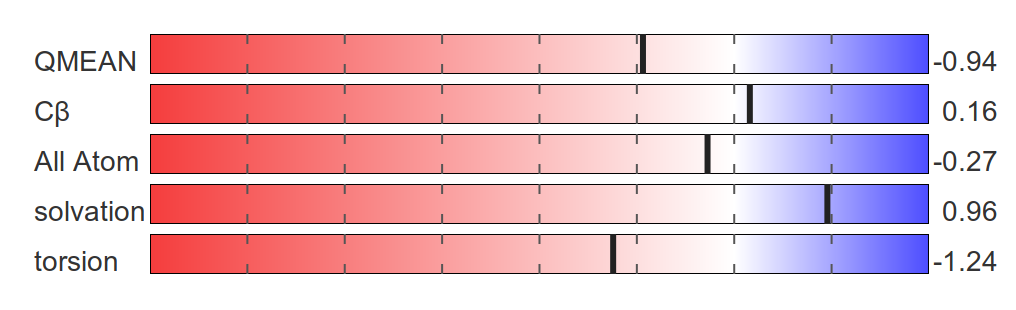

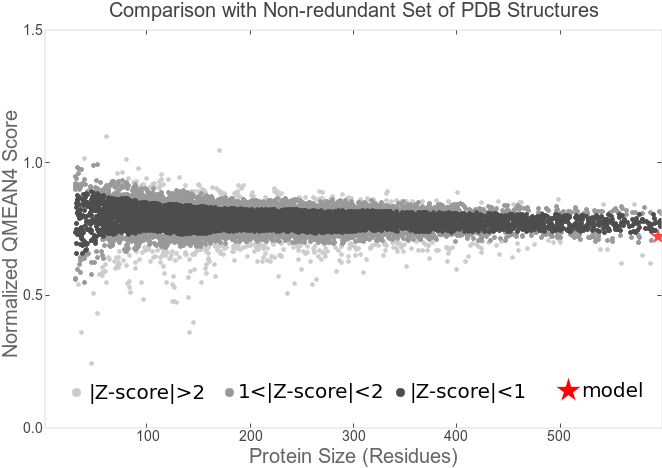

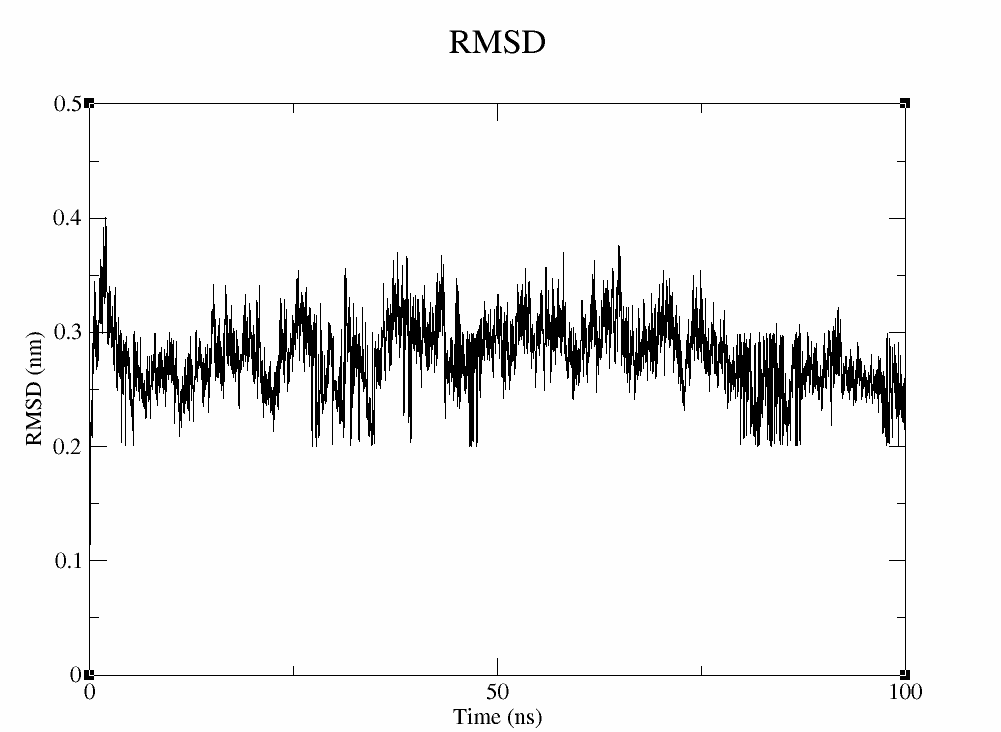

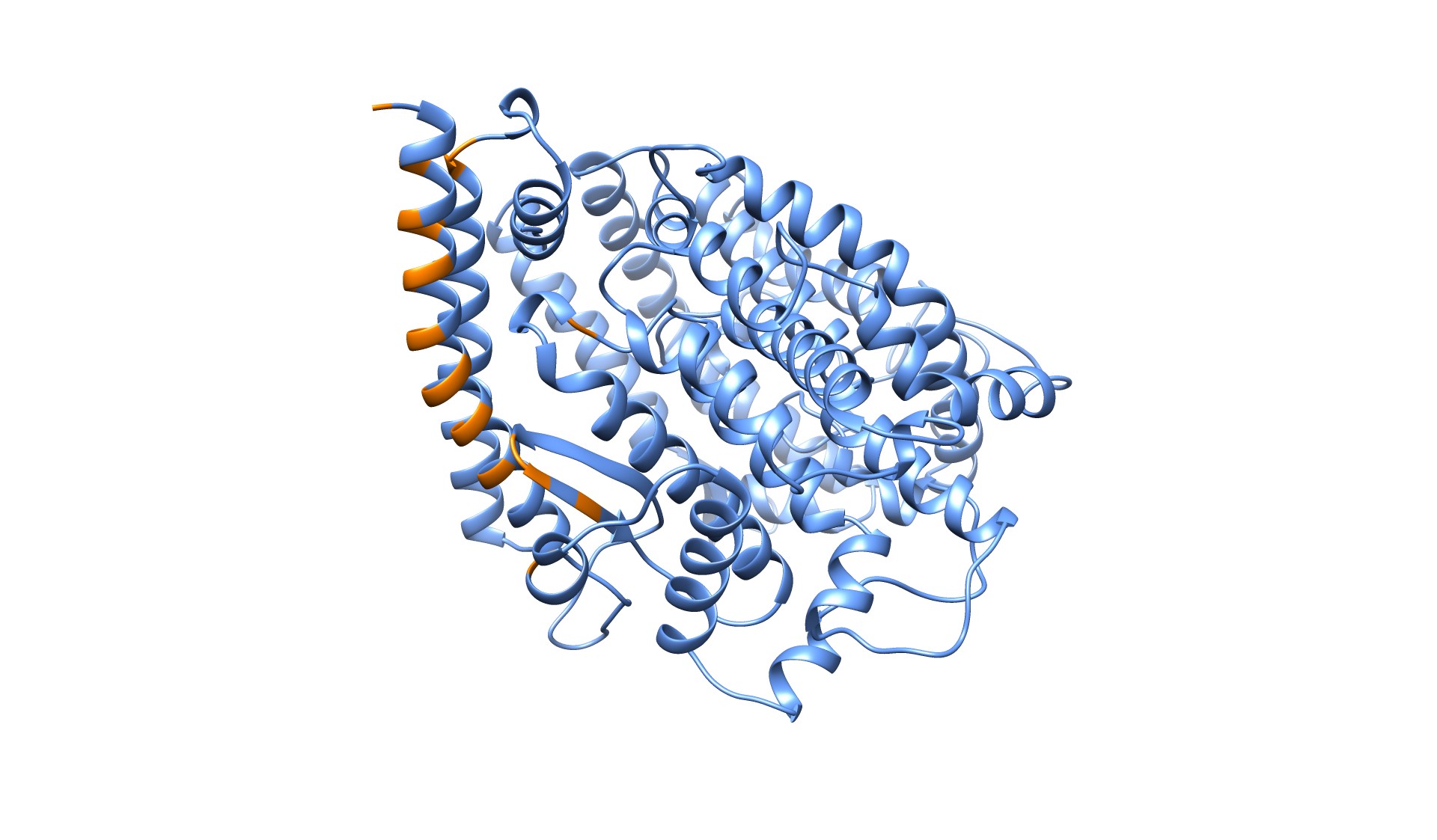


**DOG ACE2**


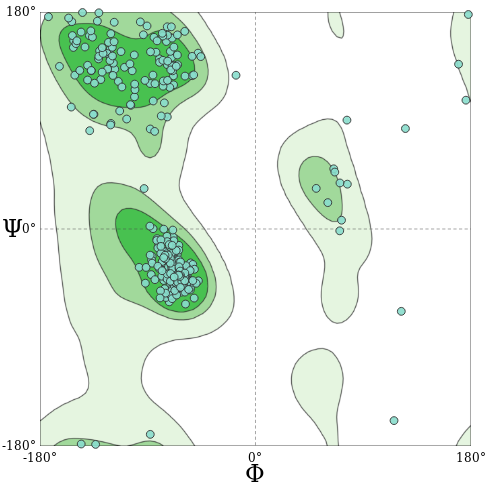

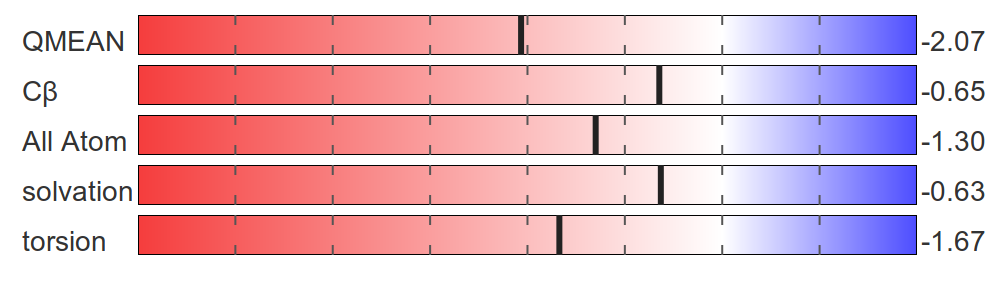

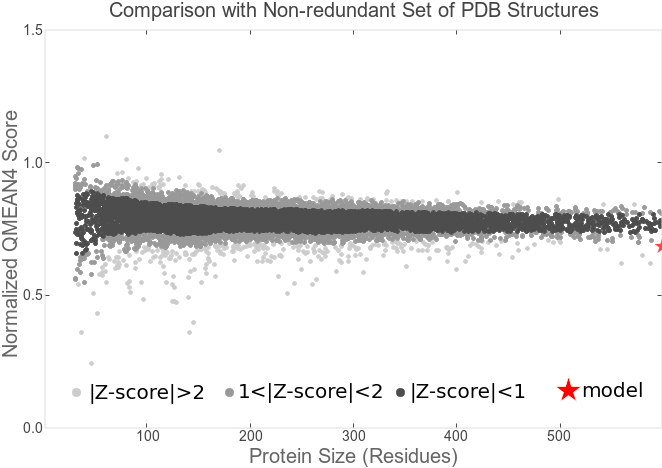

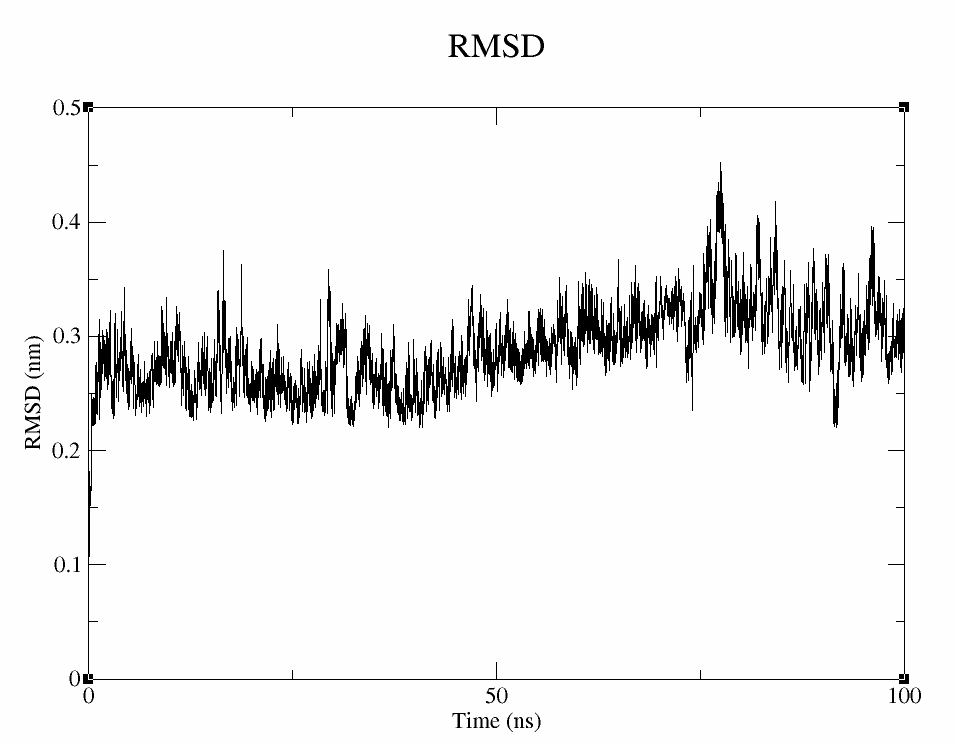

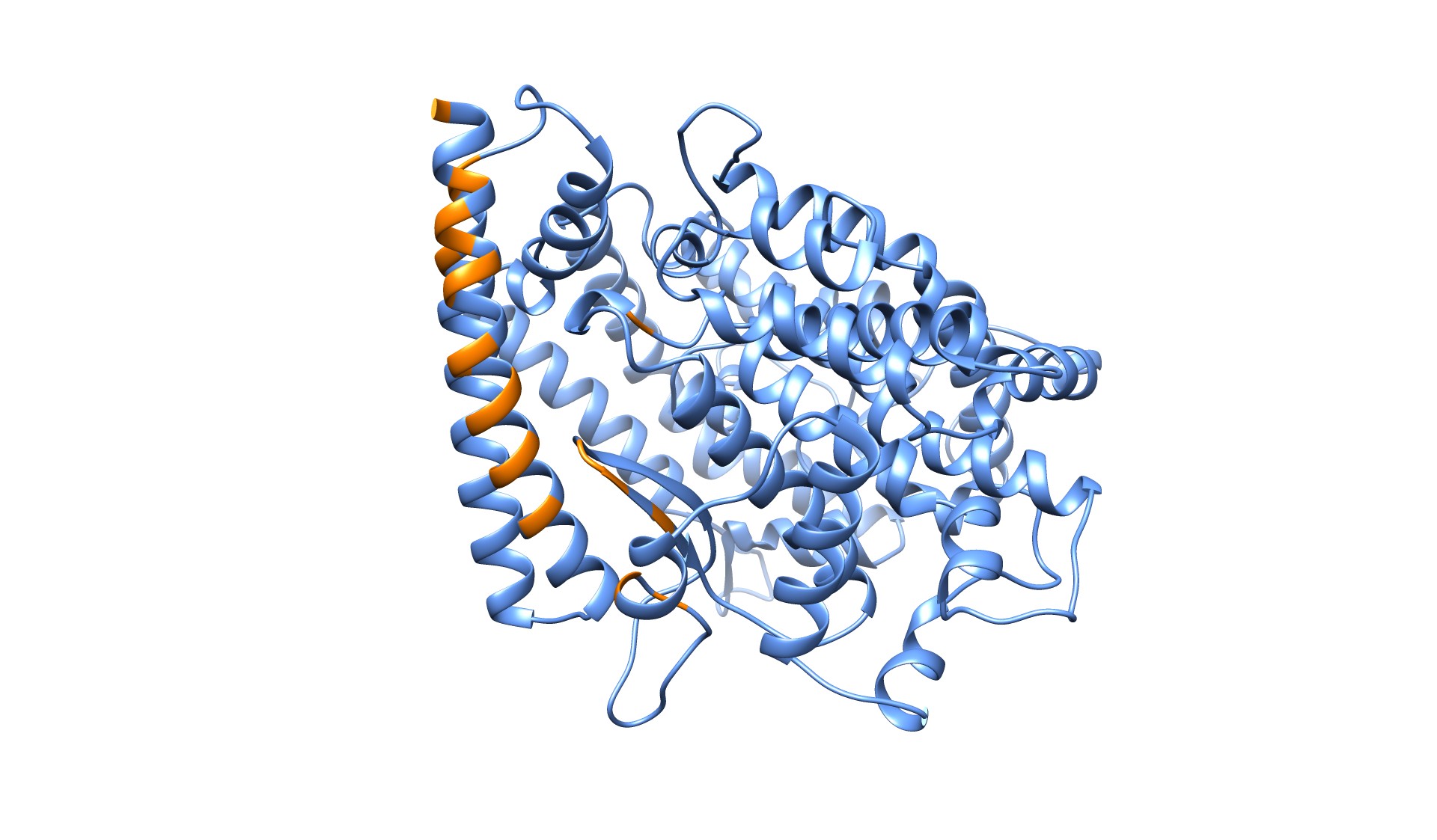


**FERRET ACE2**


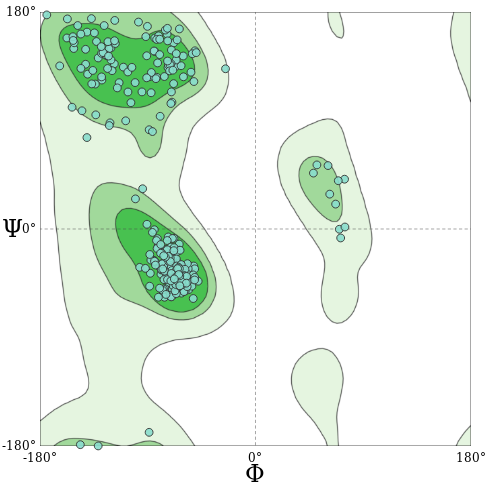

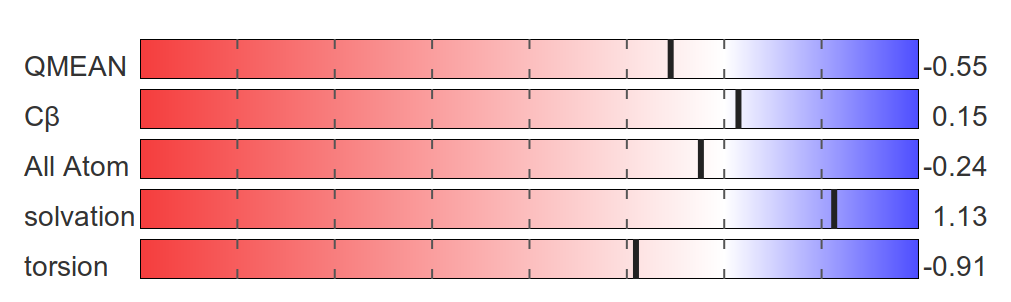

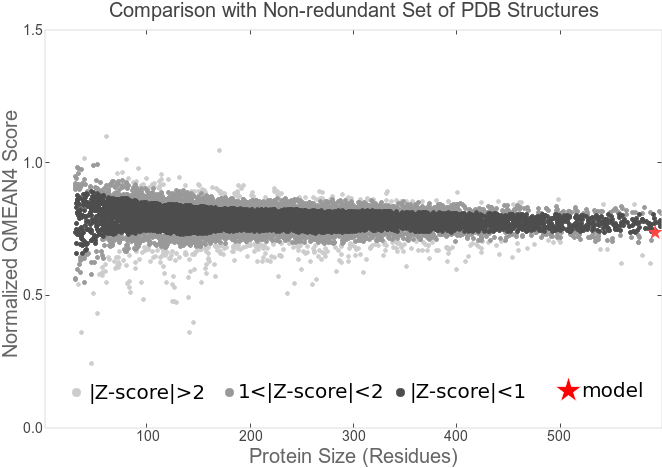

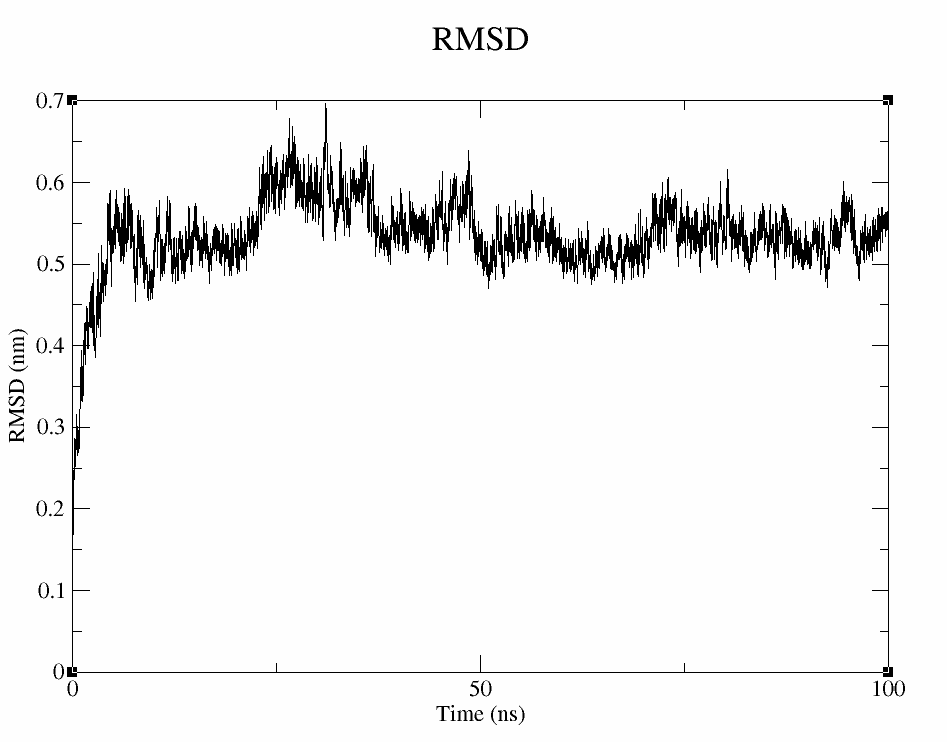

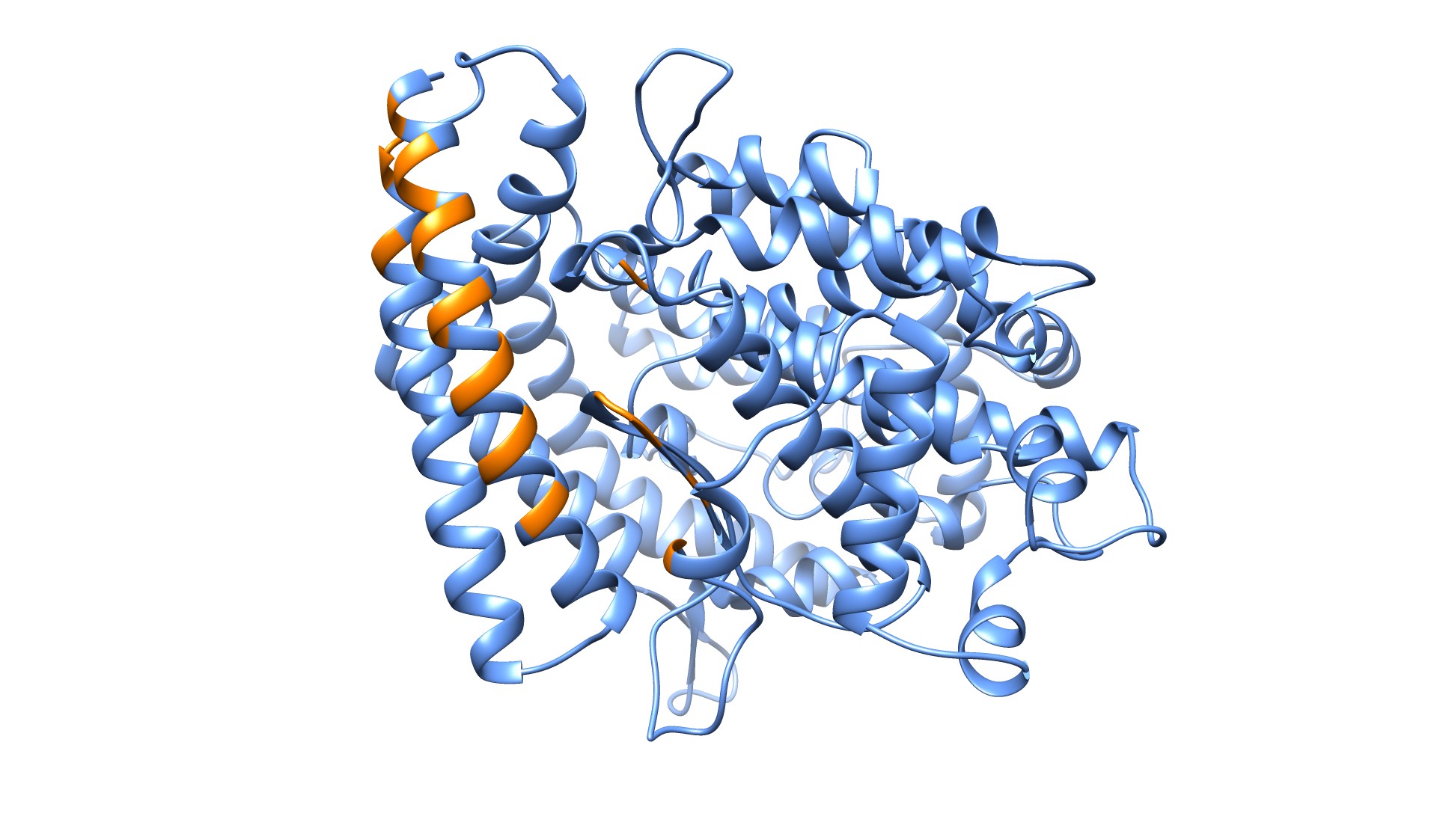


**HAMSTER ACE2**


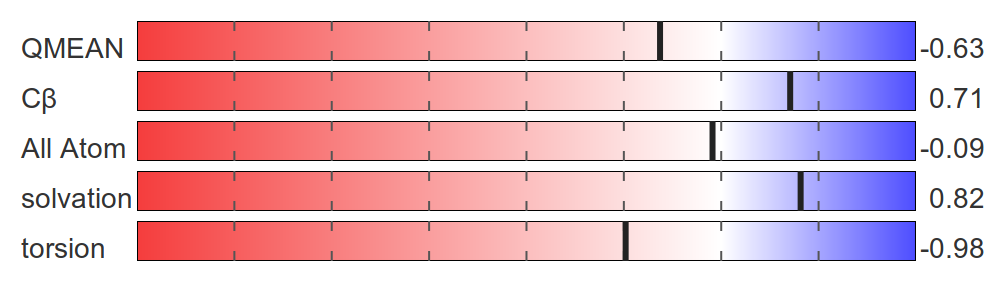

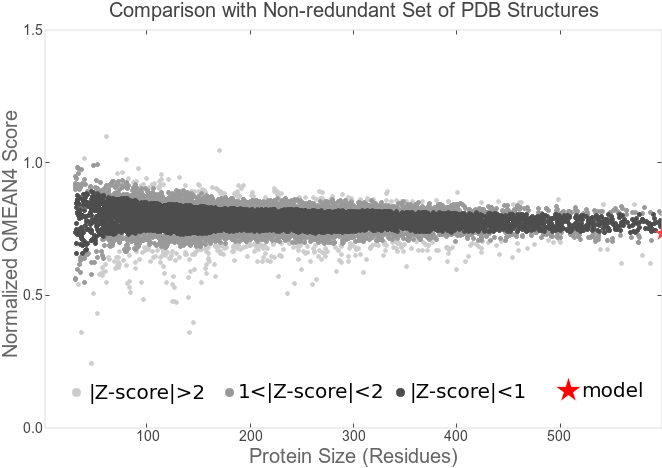

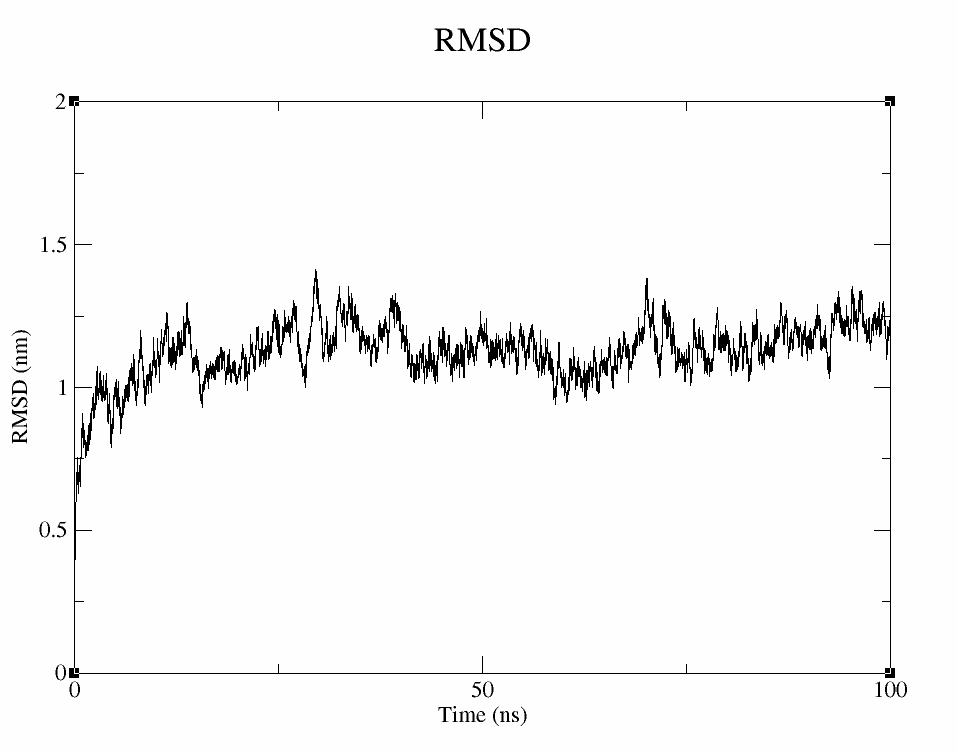

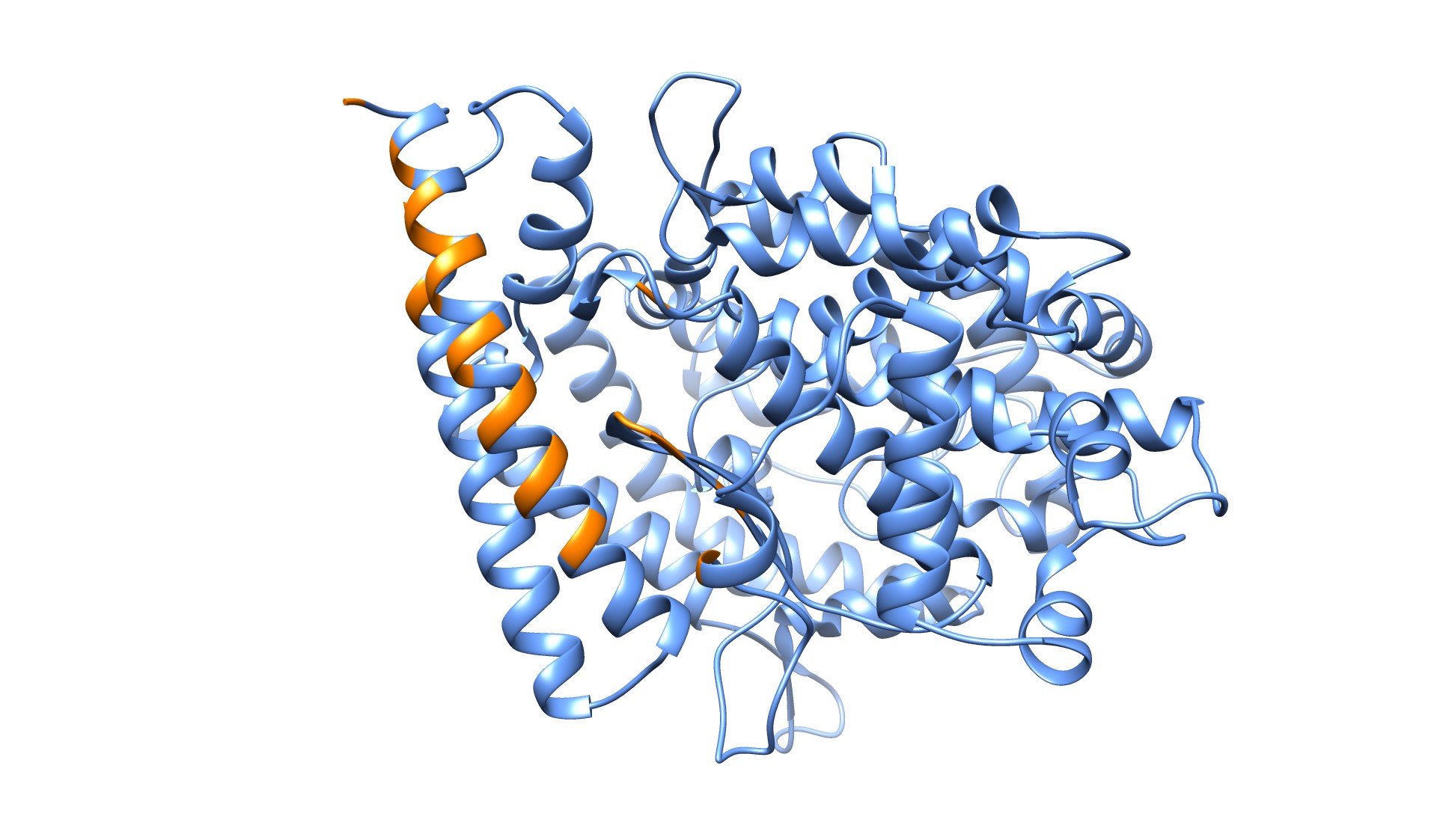


**
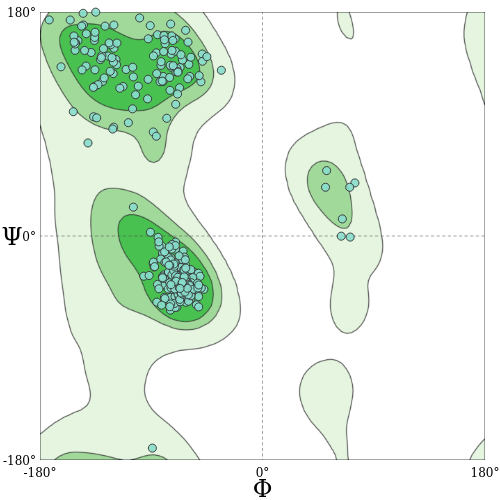
**

**MONKEY ACE2**

**
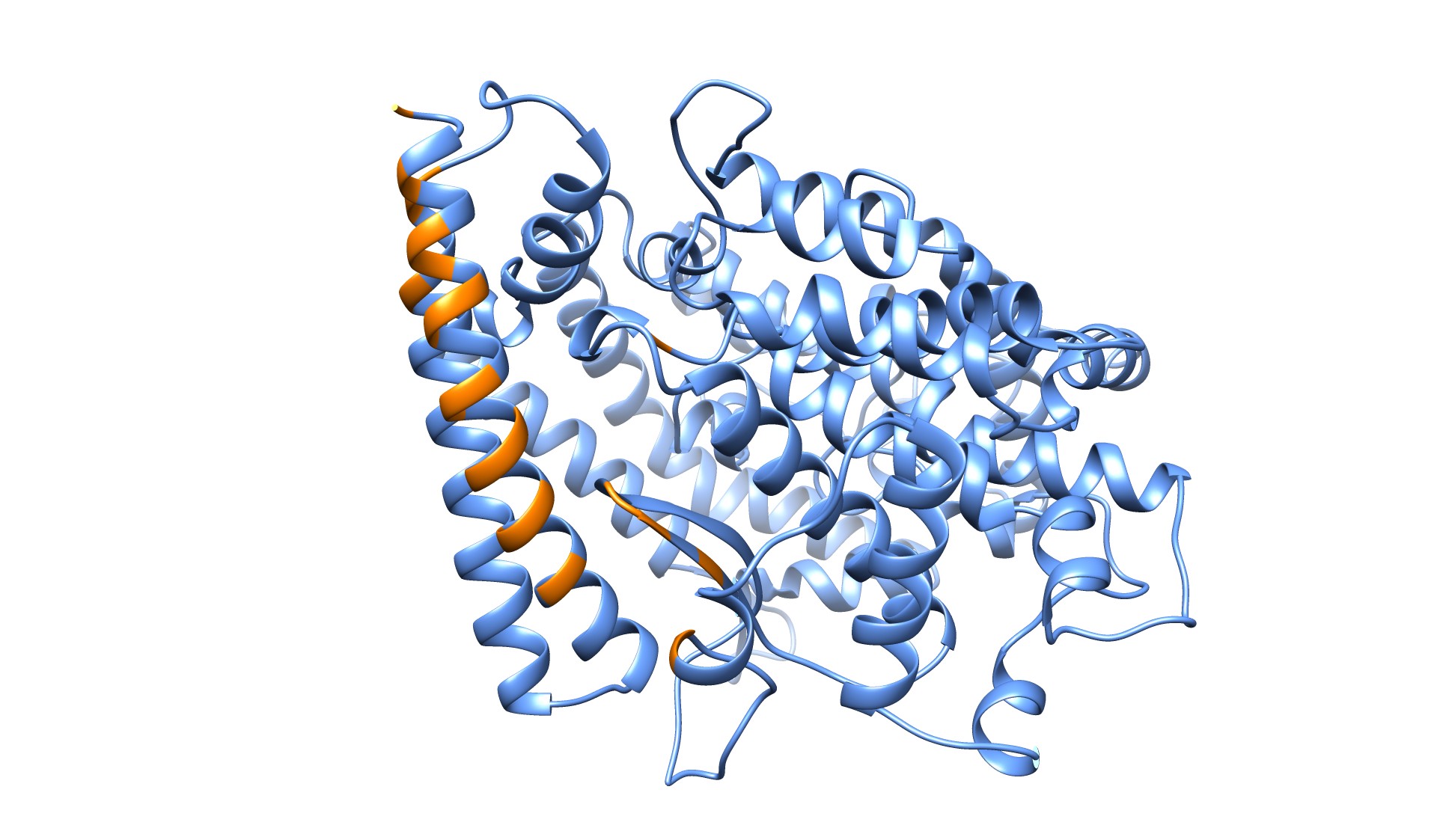

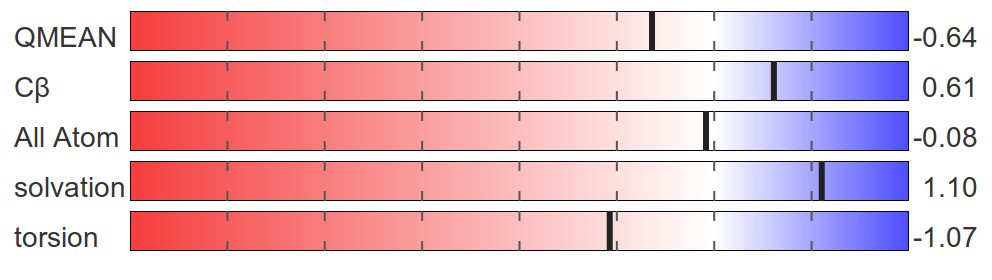
**


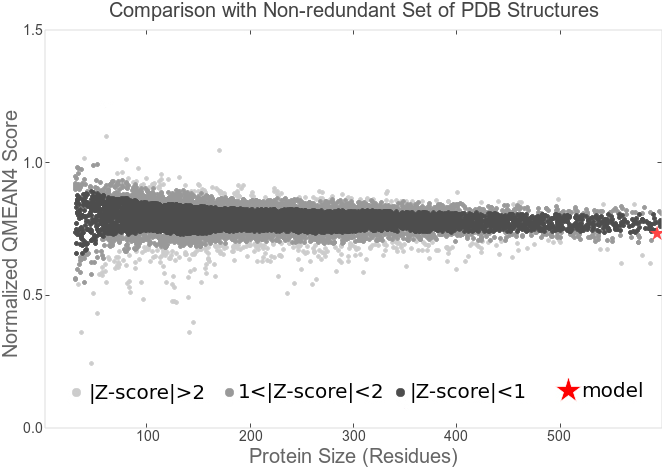

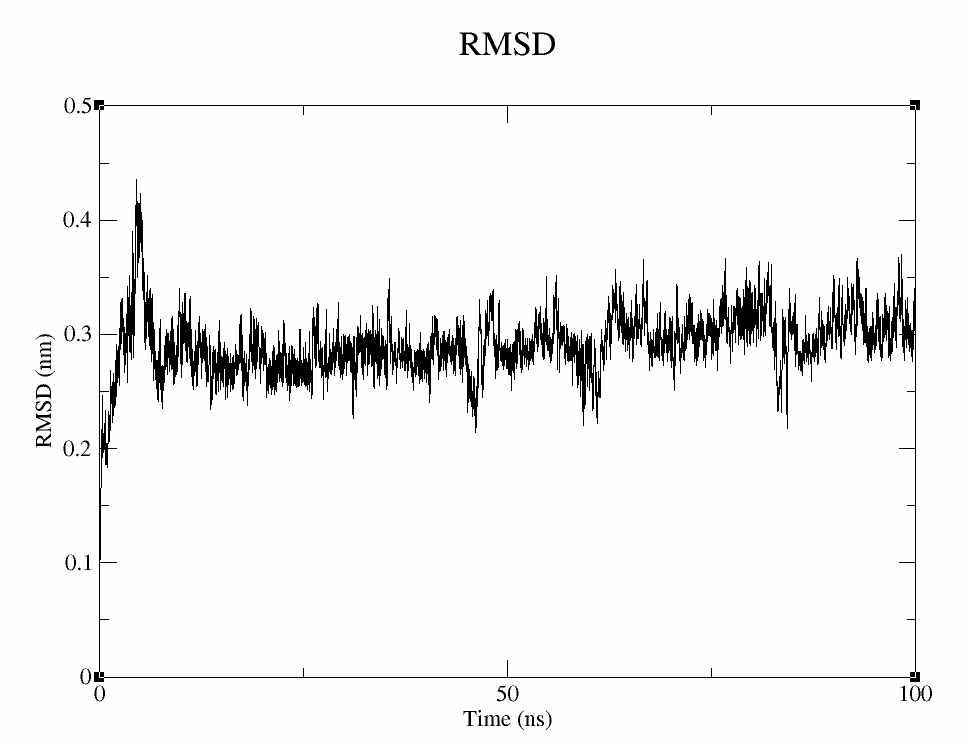


**
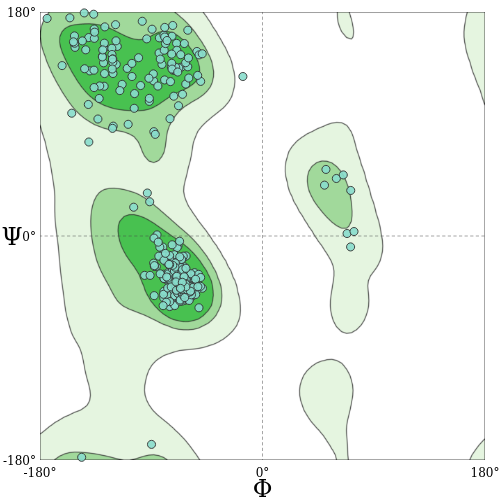
**

**MOUSE ACE2**


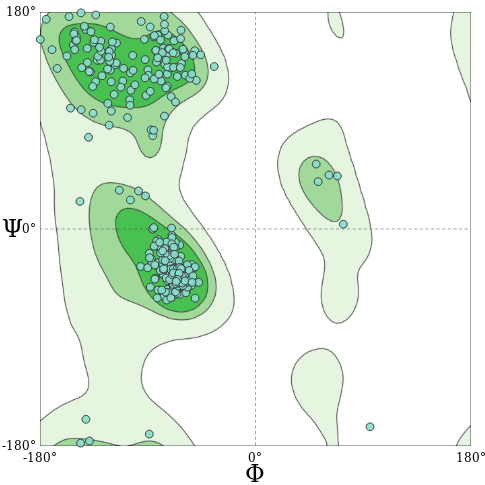

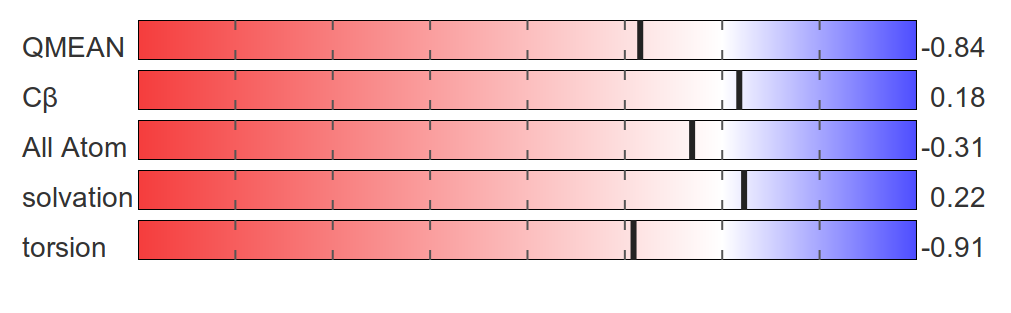

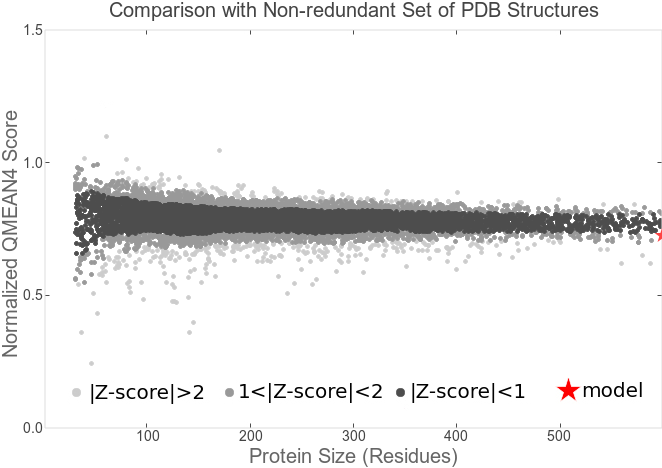

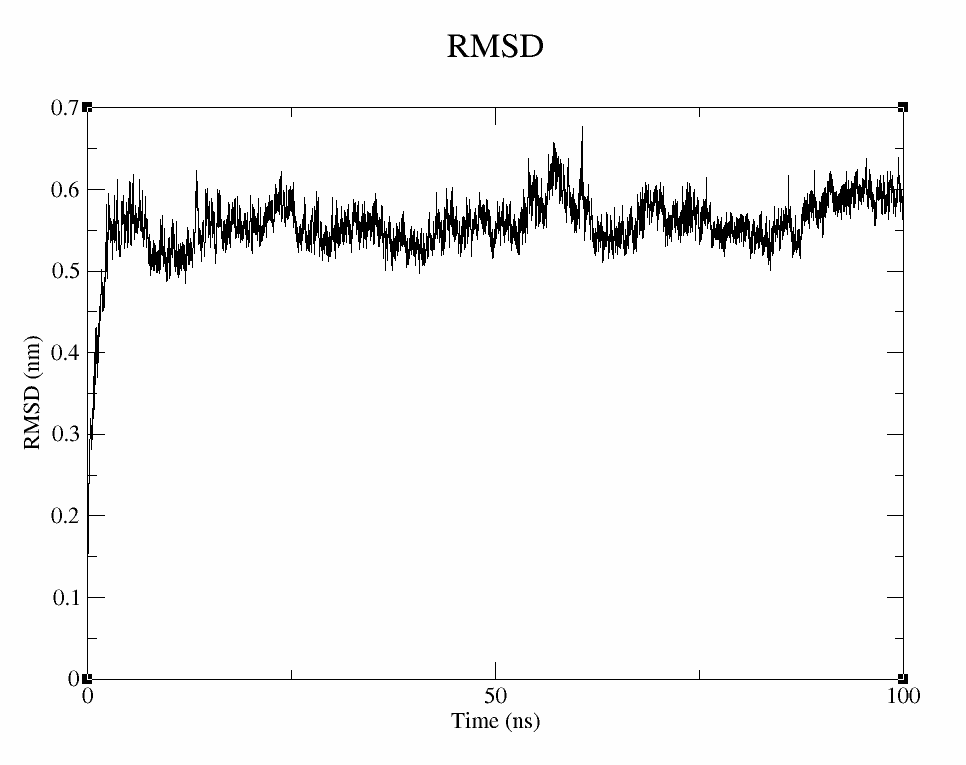

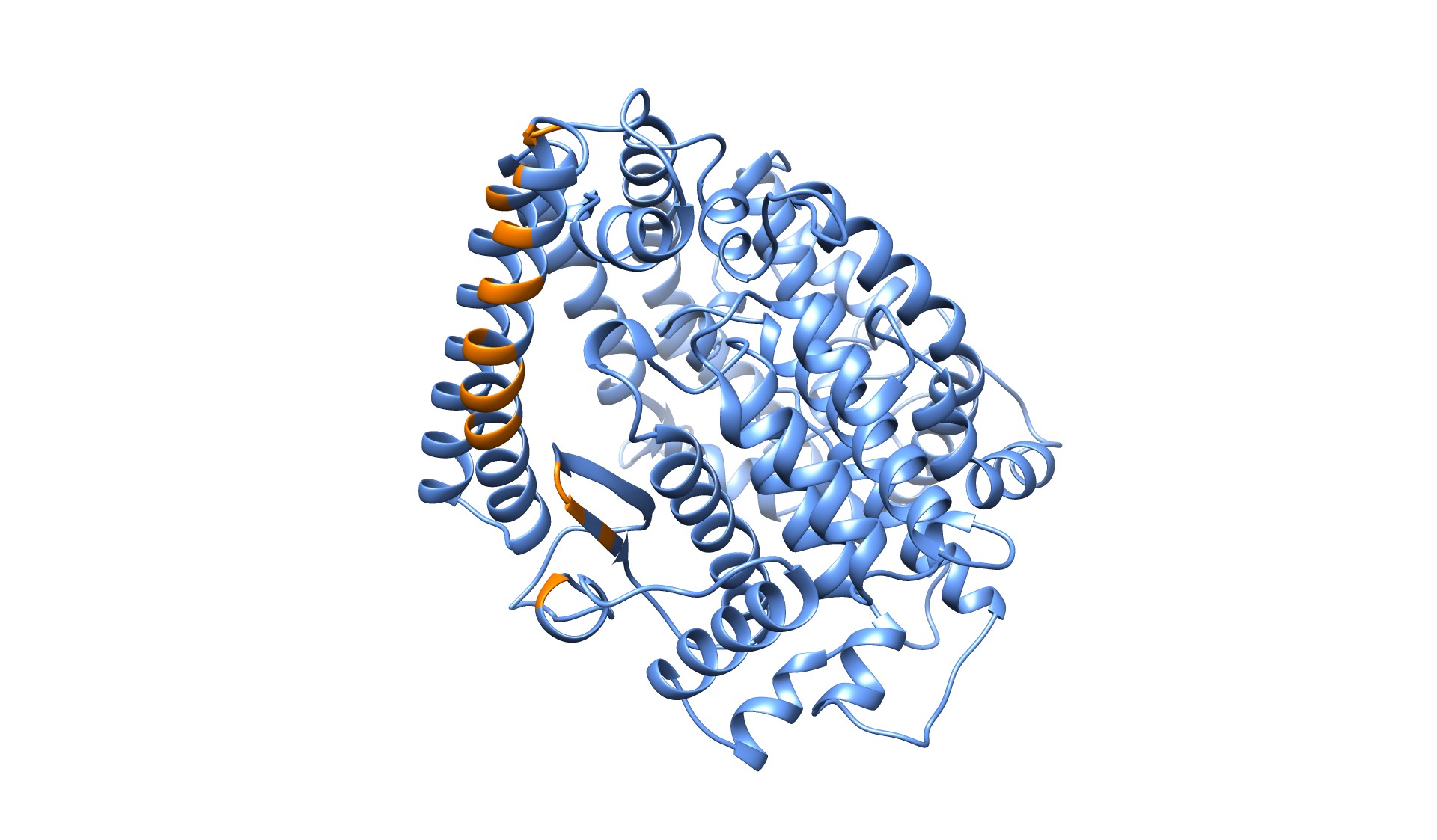


**PANGOLIN ACE2**

**
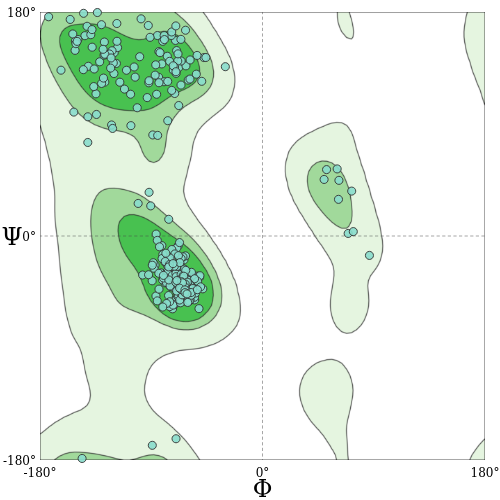
**


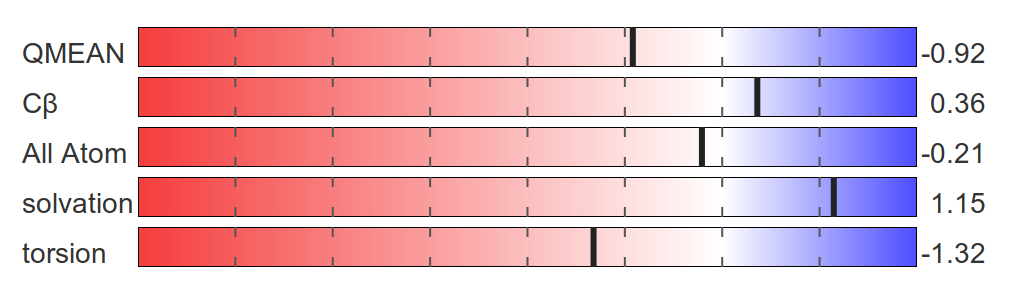

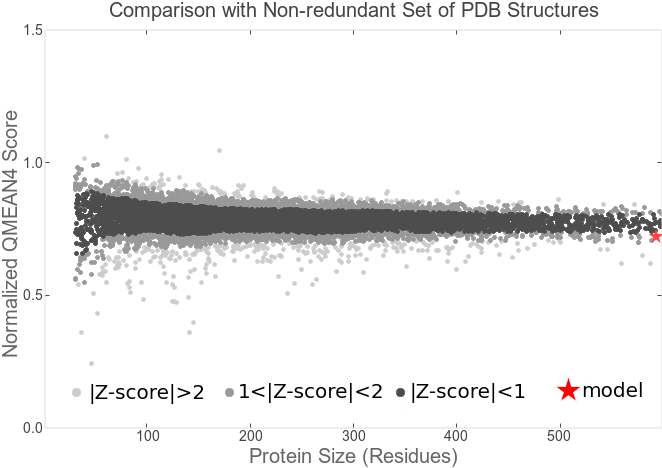

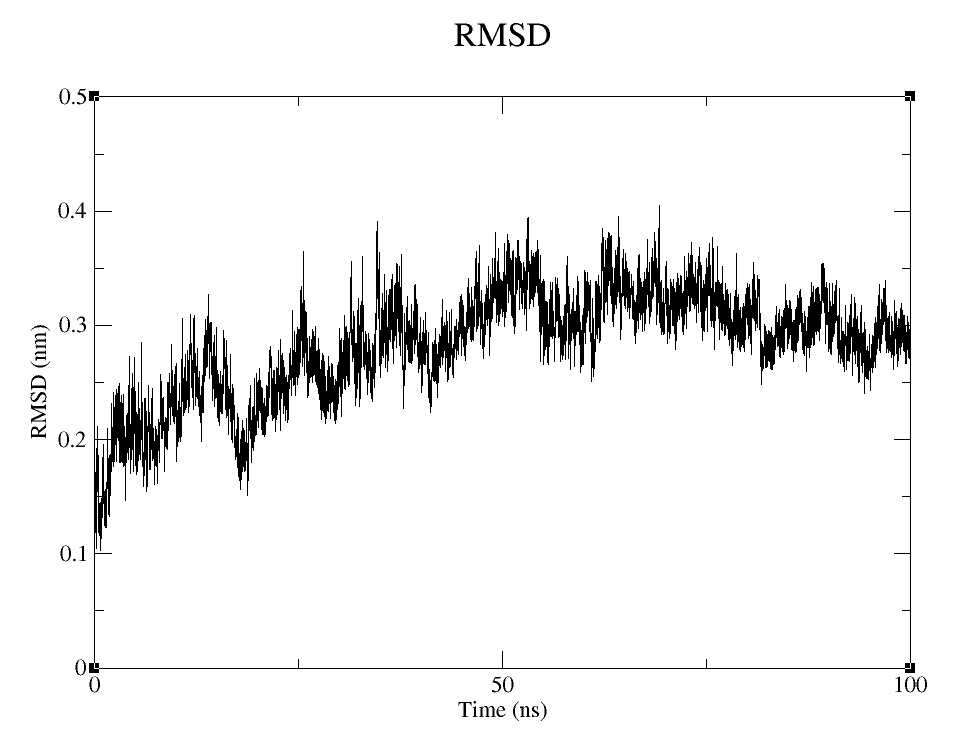

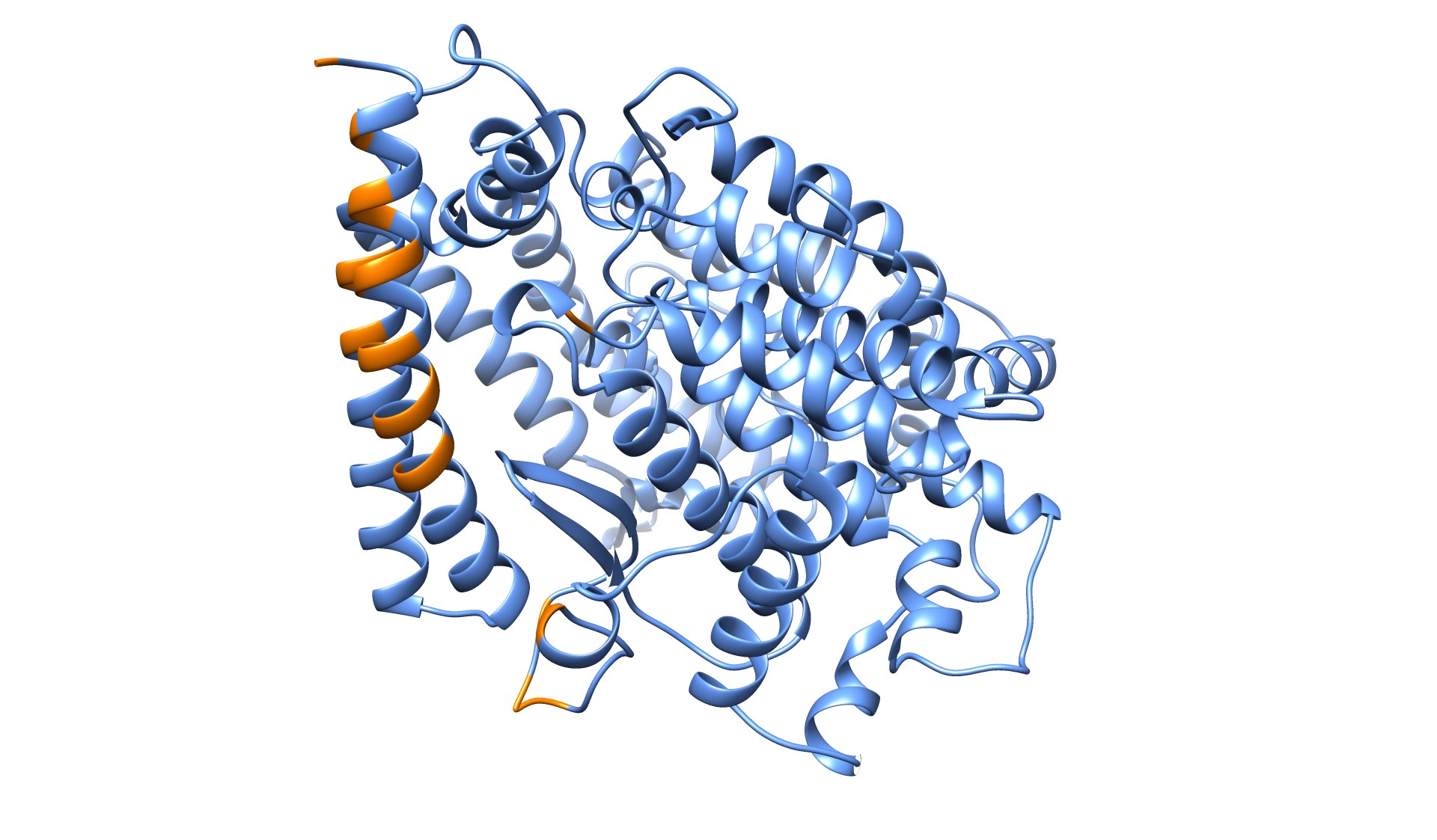


**SNAKE ACE2**


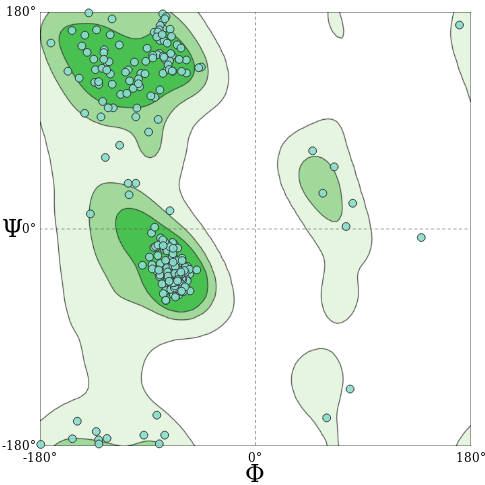

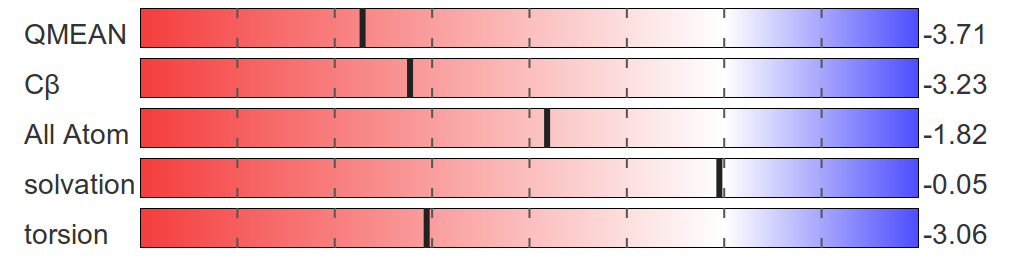

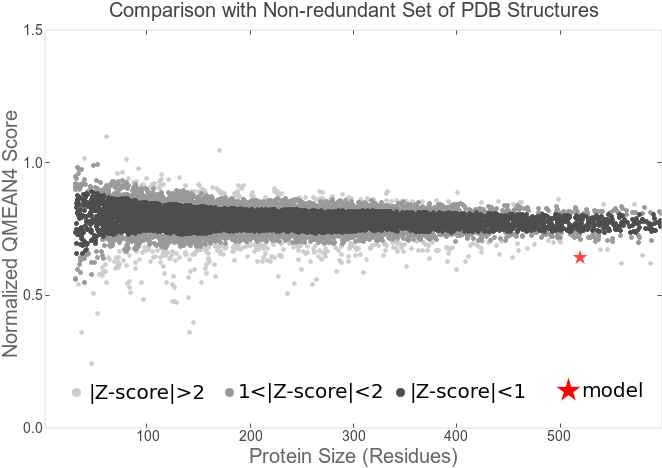

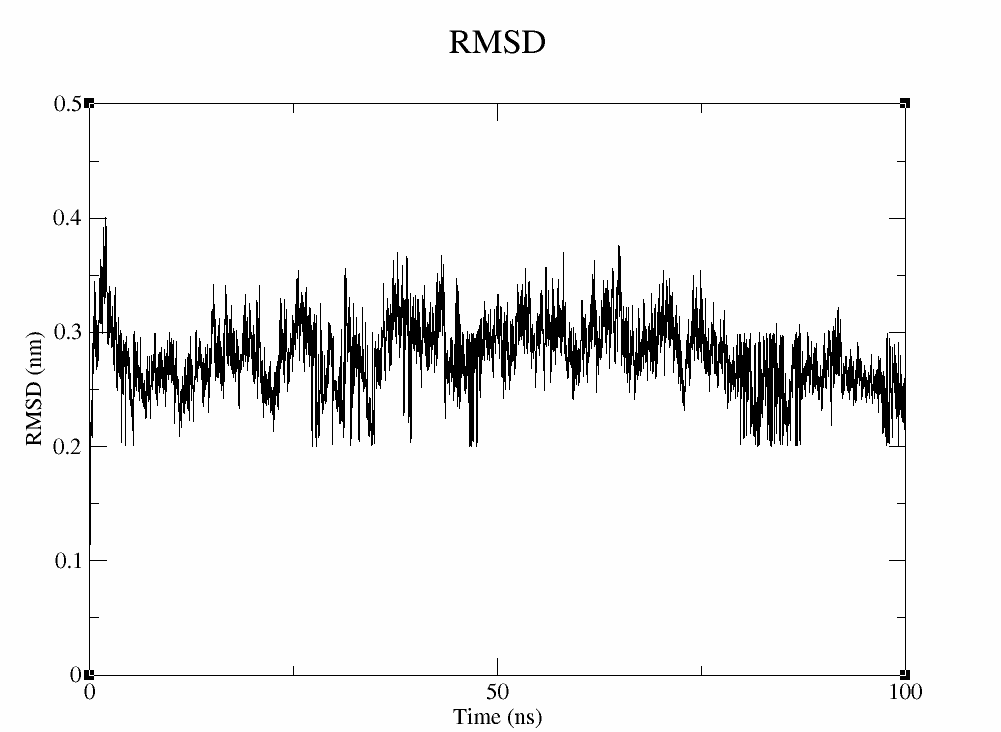

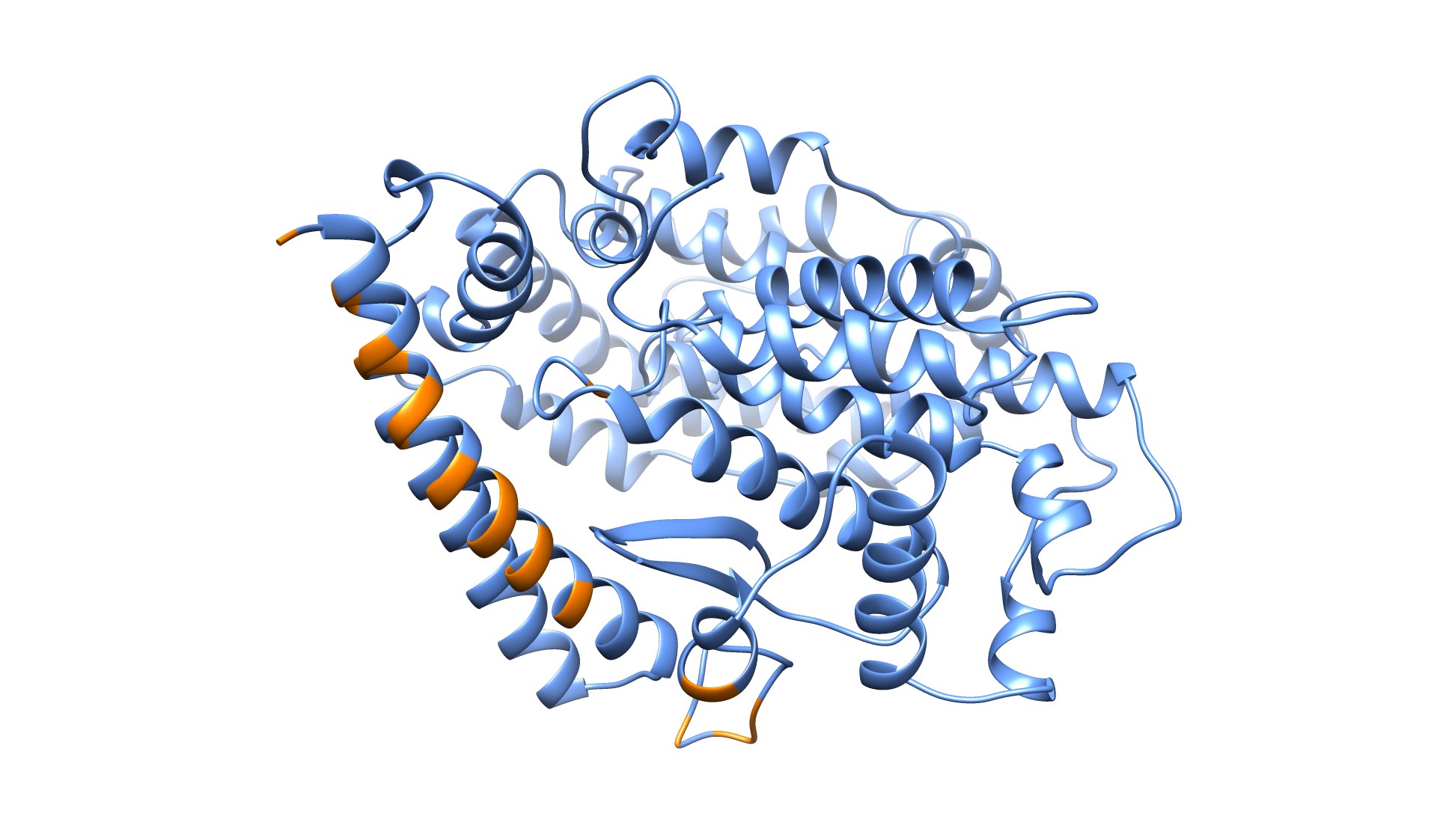


**HORSE ACE2**


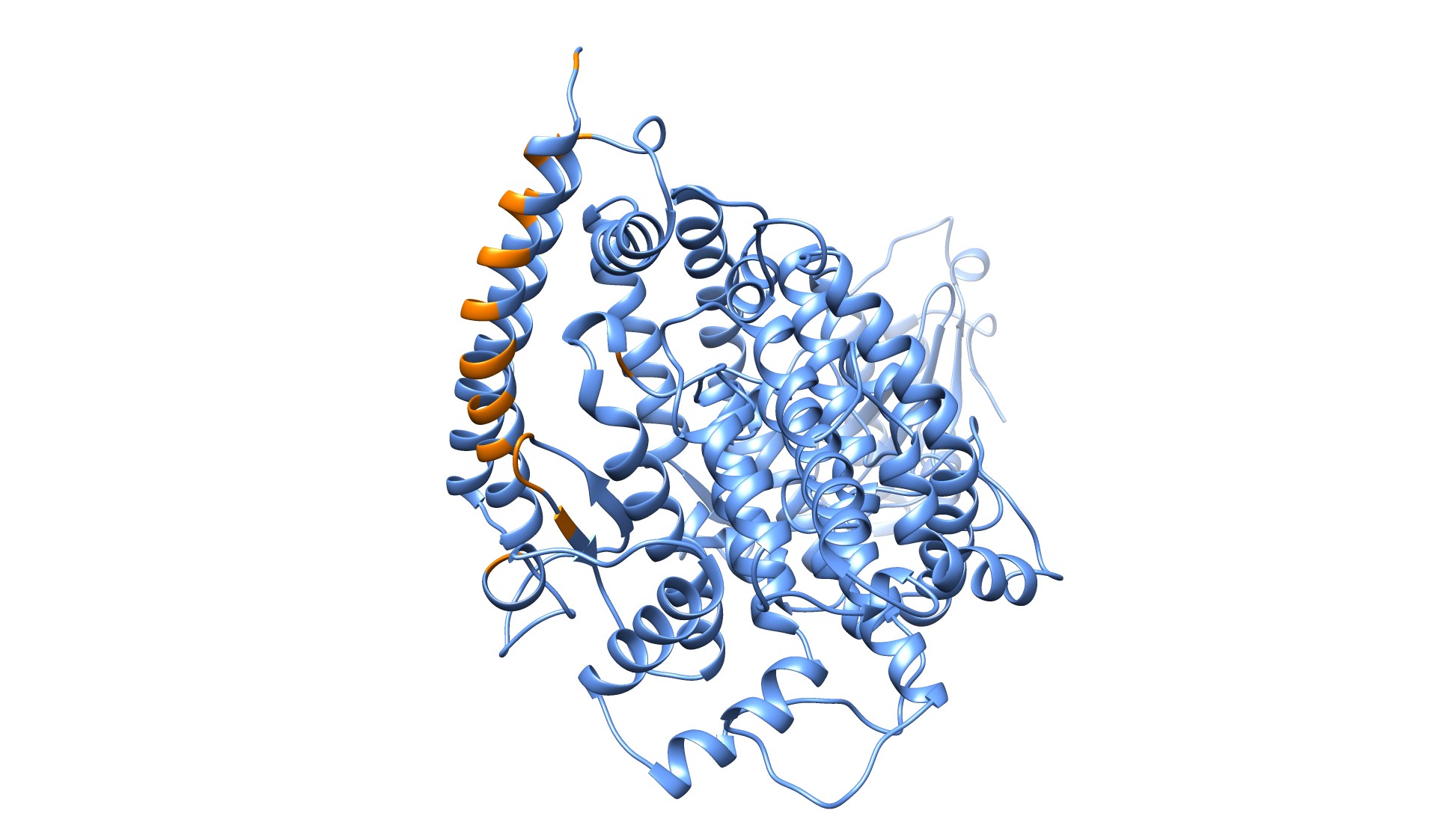

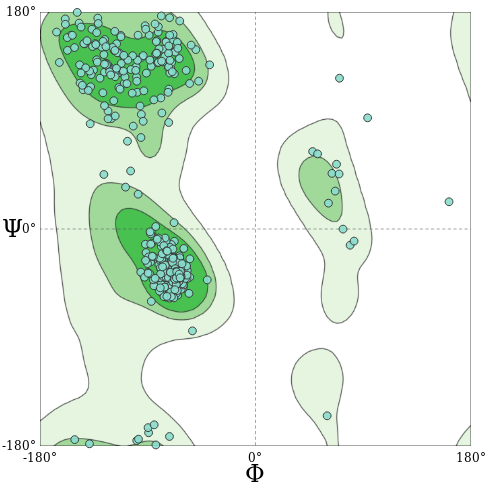

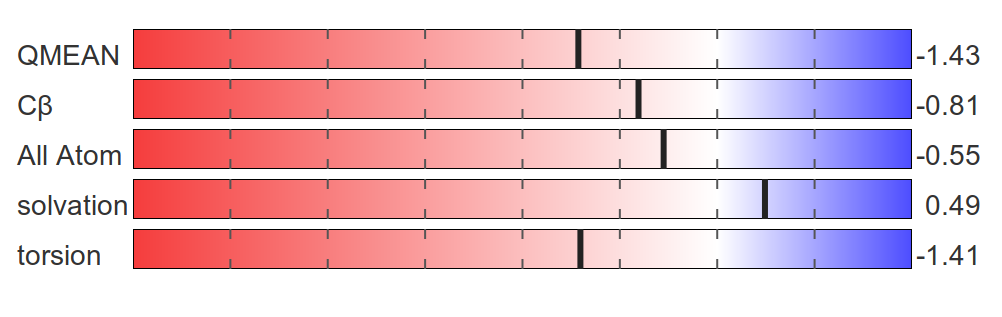

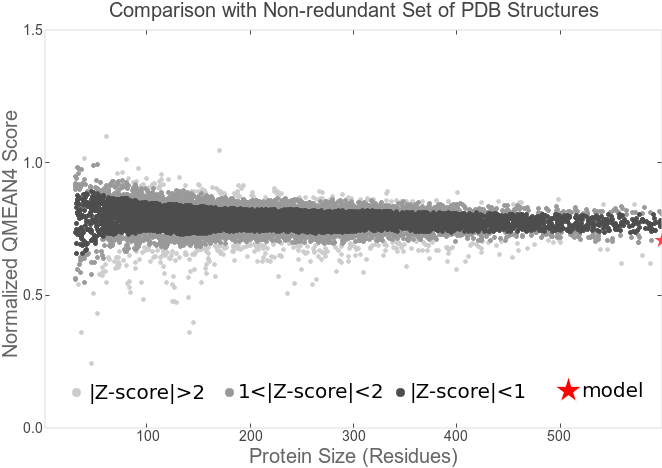

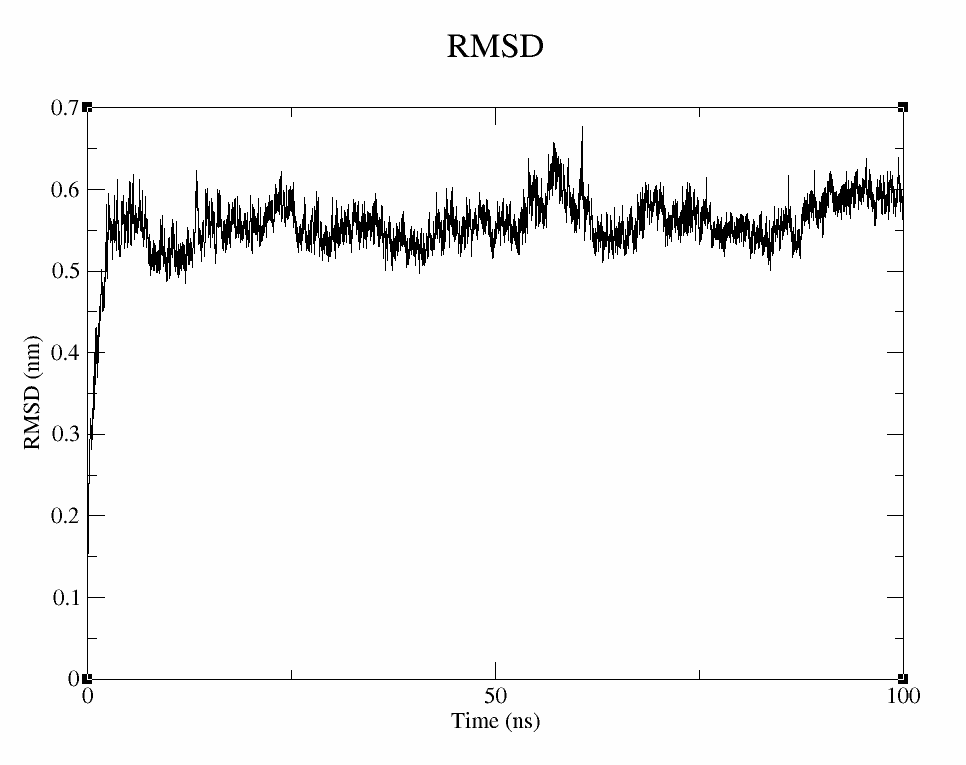


**BOVINE(COW) ACE2**


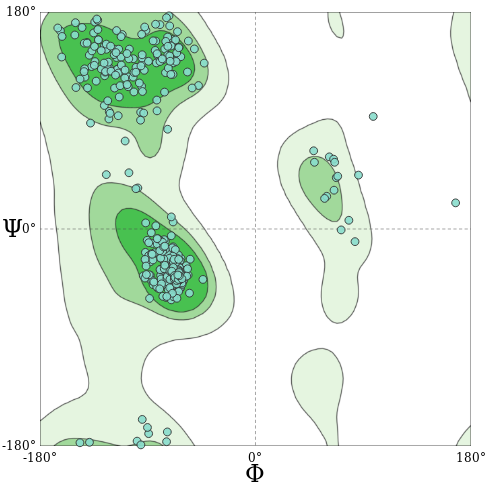

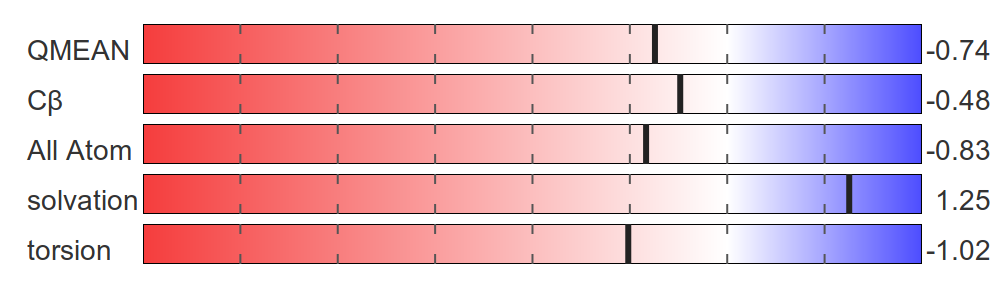

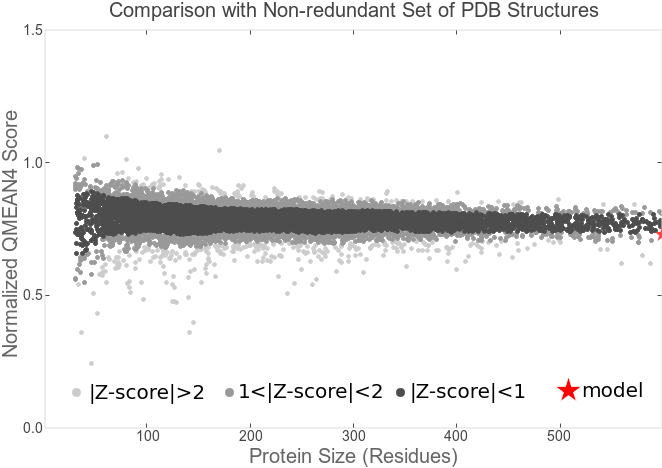

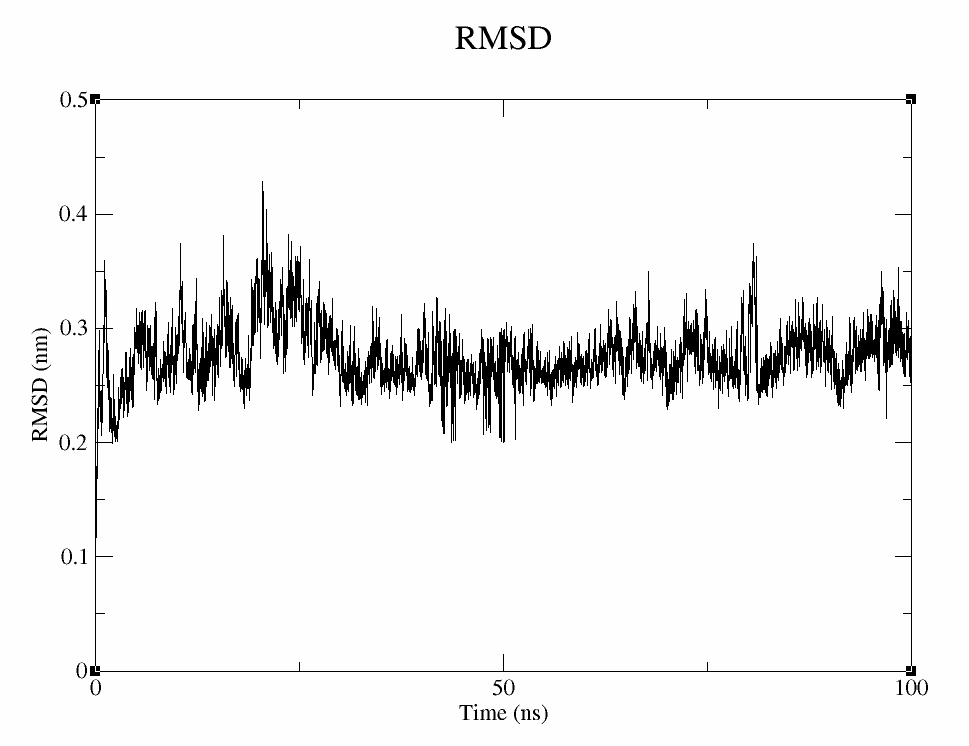

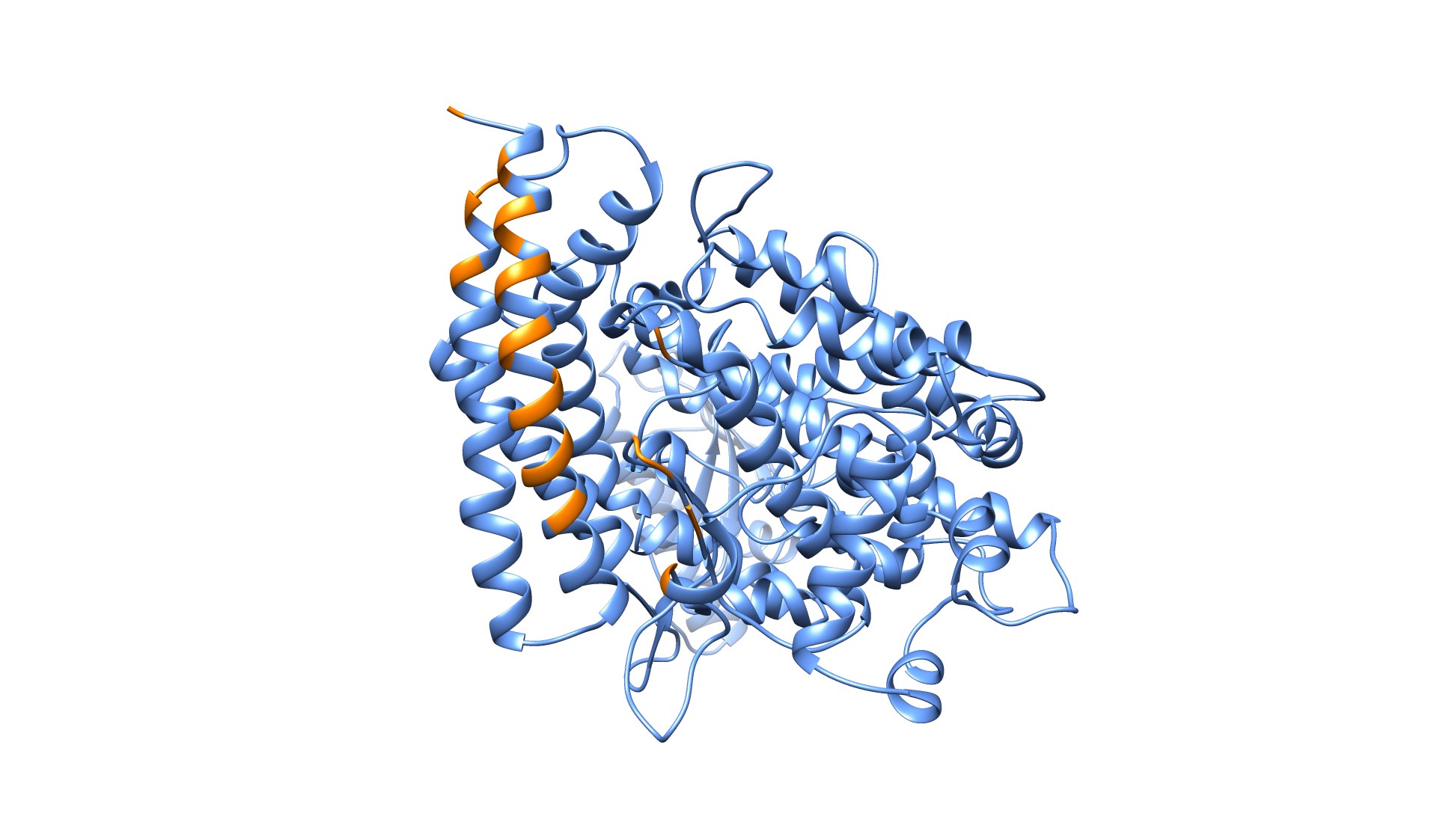


**TIGER ACE2**


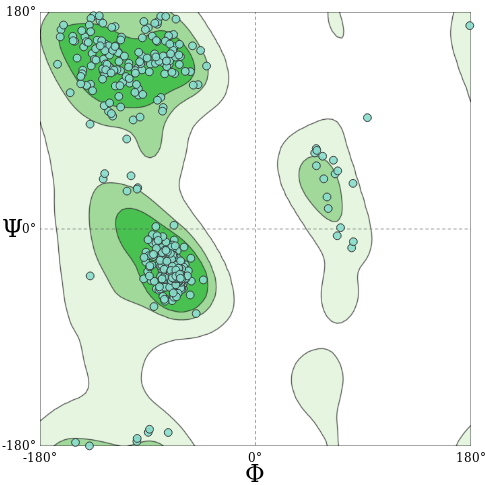

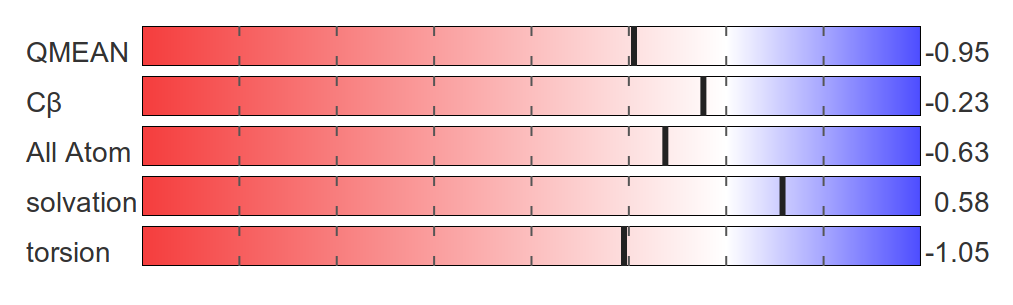

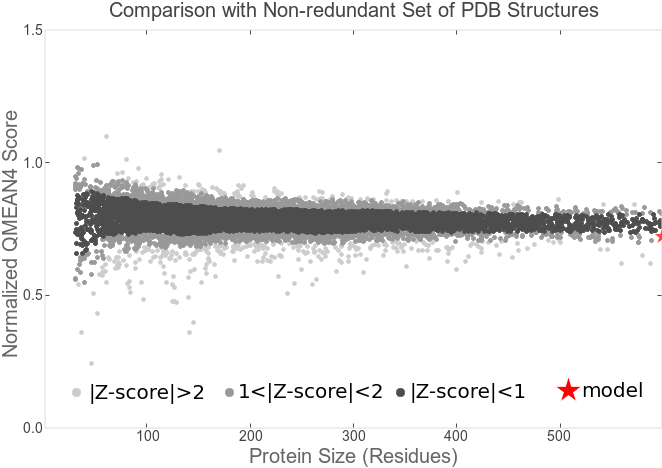

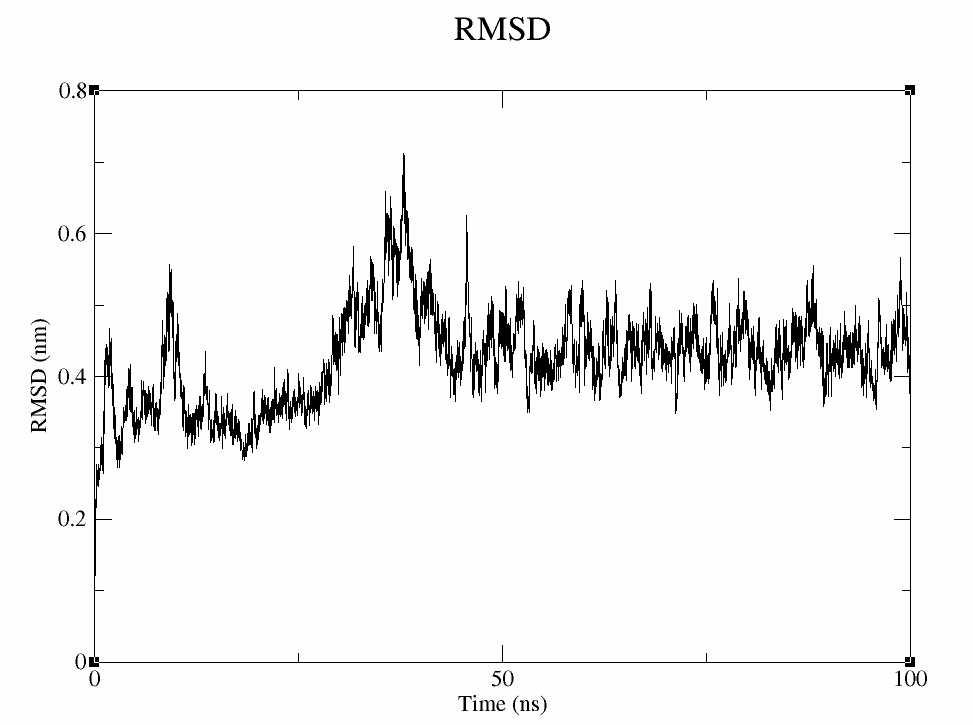

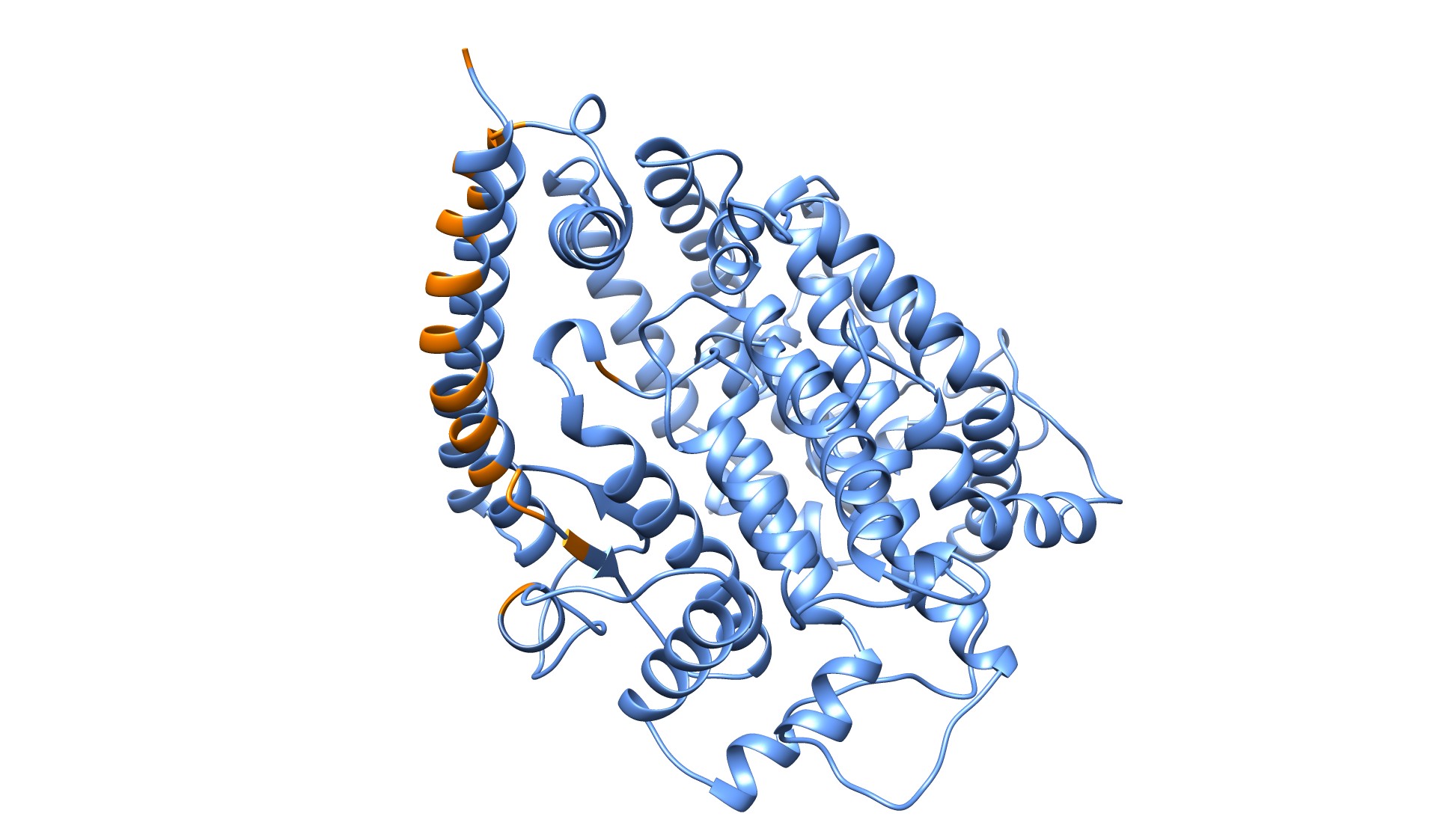


**Supplementary Figure 4**. Modelled ACE2 structures for selected species (blue is full ACE2 structure; orange denotes residues interacting with S protein RBD), together with Ramachandran plots, molecular dynamics RMSD plots, and quality metrics


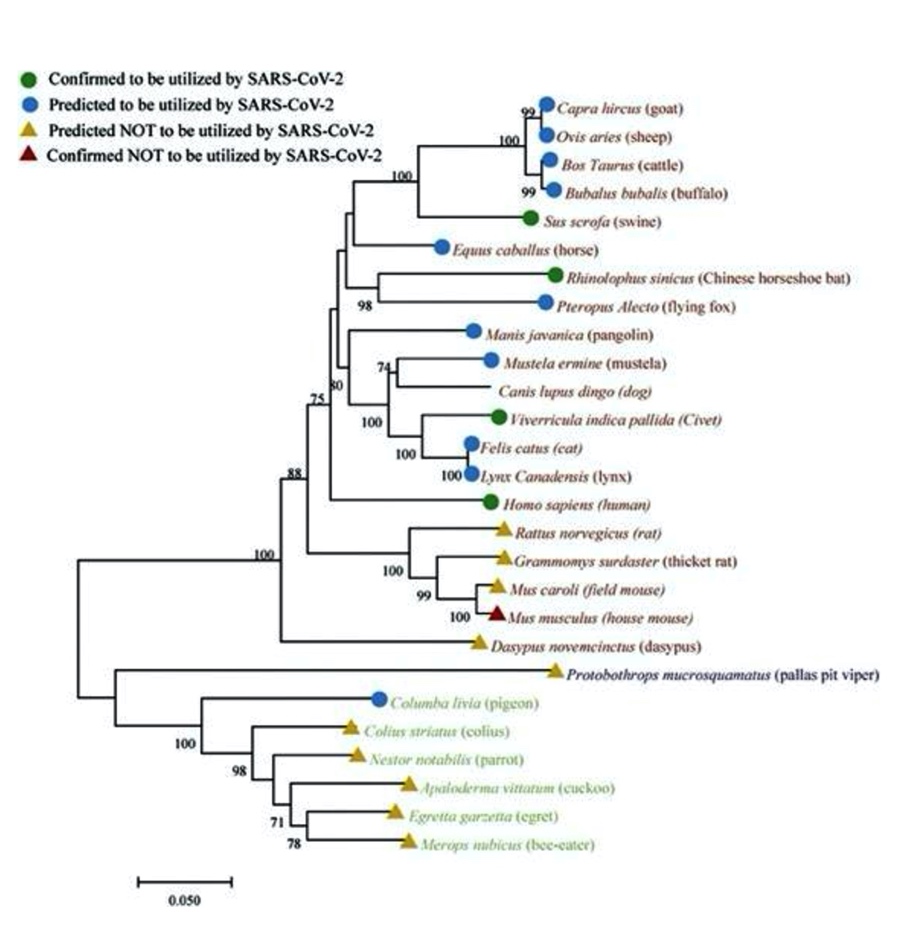


**Supplementary Figure 5**. Predicted and confirmed utilization of ACE2 receptors by the SARS-Cov-2 spike protein based on sequence homology from Qui et al.^1^

**References**

1 Qiu, Y. *et al.* Predicting the angiotensin converting enzyme 2 (ACE2) utilizing capability as the receptor of SARS-CoV-2. *Microbes Infect.* **22**, 221-225, doi:10.1016/j.micinf.2020.03.003 (2020).
